# *Dracaena trifasciata* and *Dracaena angolensis*: Phytochemistry, Pharmacological Activities, and Mechanistic Insights

**DOI:** 10.3390/molecules31193520

**Published:** 2026-10-02

**Authors:** Kinga Kedzierska, Agnieszka Welman-Styk, Grzegorz Adamczuk, Anna Rymuszka

**Affiliations:** Department of Physiology and Toxicology, Faculty of Medicine, The John Paul II Catholic University of Lublin, Konstantynów Str. 1i, 20-708 Lublin, Poland; kinga.kedzierska@kul.pl (K.K.); agnieszka.welman-styk@kul.pl (A.W.-S.); grzegorz.adamczuk@kul.pl (G.A.)

**Keywords:** *Dracaena trifasciata*, *Dracaena angolensis*, phytochemistry, steroidal saponins, homoisoflavonoids, pharmacological activity, molecular mechanisms, bioactive compounds

## Abstract

*Dracaena trifasciata* and *Dracaena angolensis* (Asparagaceae) are medicinal plants traditionally used in Africa and Asia for inflammatory disorders, infections and other conditions. This review critically synthesizes current knowledge on their phytochemistry, biological activities, safety, and underlying molecular mechanisms. A structured literature search was conducted in Scopus, Web of Science and PubMed, supplemented by citation tracking. The final evidence synthesis comprised 85 primary studies, with review articles and contextual sources used separately for background and interpretation. Both species contain structurally diverse metabolites, predominantly steroidal saponins, flavonoids, and homoisoflavonoids, together with phenolic acids, terpenoids and other steroidal glycosides. Experimental studies document chemical antioxidant capacity, anti-inflammatory and antimicrobial effects, enzyme-inhibitory and metabolic activities, and in vitro cytotoxic or antiproliferative effects, although evidence strength varies by endpoint and preparation. Mechanistic evidence includes Nrf2/ARE-associated responses, NF-κB-related signalling, apoptosis and cell-cycle regulation, and quorum-sensing-associated virulence effects, whereas several proposed molecular targets remain supported only by computational analyses. Most studies rely on crude or partially characterized extracts, and safety evidence remains limited to a small number of acute and 28-day repeated-dose studies. Bioactivity-guided isolation, compound-level mechanistic validation, pharmacokinetic characterization and more comprehensive toxicological studies are therefore required before translational potential can be established.

## 1. Literature Review Methodology

This article was designed as a critical narrative review supported by a structured and reproducible literature search. The methodological quality and reporting of the review were considered with reference to the Scale for the Assessment of Narrative Review Articles (SANRA) [1]. The structured search was used to improve the transparency, reproducibility, and comprehensiveness of literature identification and study selection rather than to conduct a formal systematic review.

A comprehensive literature search was conducted in the Scopus, Web of Science Core Collection, and PubMed databases from database inception to 14 September 2026. The search strategy combined the accepted taxonomic names of the investigated species (*Dracaena trifasciata* and *Dracaena angolensis*) and their historically used synonyms (*Sansevieria trifasciata* and *Sansevieria cylindrica*) with terms related to phytochemistry, pharmacological and biological activity, molecular mechanisms, toxicology and safety, and ethnobotany and ethnopharmacology. No restrictions based on publication year, document type, language, or full-text availability were applied at the database-search stage. In addition, reference lists of eligible primary studies and relevant review articles were manually screened to identify potentially eligible primary studies not retrieved through the database searches.

To improve the comprehensiveness of the search, five complementary search modules were applied: Module A (Phytochemistry), Module B (Pharmacological and Biological Activity), Module C (Molecular Mechanisms), Module D (Toxicology and Safety), and Module E (Ethnobotany and Ethnopharmacology). The complete database-specific search strategies, including all search terms, Boolean operators, and search fields used in PubMed, Scopus, and Web of Science Core Collection, are provided in Supplementary Table S2.

Original research articles were eligible for inclusion in the primary evidence synthesis when they reported directly attributable data for *D. trifasciata* and/or *D. angolensis* in at least one of the predefined domains: phytochemistry, ethnomedicinal use, pharmacological or biological activity, molecular mechanisms, or toxicology and safety. Studies involving multiple plant species were eligible only when results for either target species could be clearly separated. Review articles and other secondary sources were not treated as independent units of evidence and were not included in the primary-study count. Selected reviews directly relevant to the target species or to specific interpretative aspects of the review (*n* = 4) were retained solely for contextual interpretation and backward citation screening and were not included in the primary-study count.

Records were excluded when they concerned non-target species without separately extractable data for either target species, lacked original data, represented secondary publications, or addressed technological, materials-science, or environmental applications outside the predefined core evidence domains. Studies of nanoparticle-based products were excluded from the primary pharmacological evidence when biological effects were attributable to the nanoparticles rather than directly to the plant preparation. Studies were not excluded solely on the basis of methodological quality; methodological limitations were instead recorded during evidence appraisal and considered when interpreting the strength of the findings.

**Figure 1:**
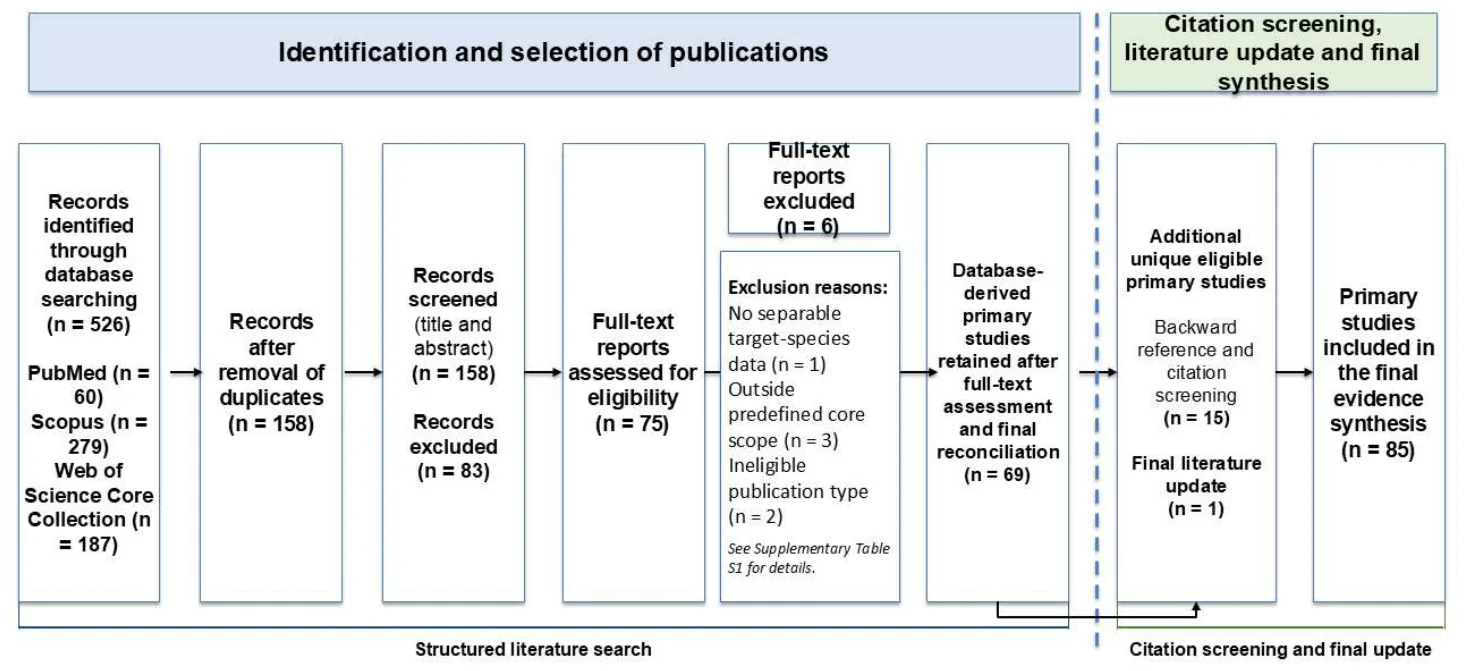
Flow diagram illustrating the literature identification and study-selection process. Database searches identified 526 records (PubMed, *n* = 60; Scopus, *n* = 279; Web of Science Core Collection, *n* = 187). After removal of 368 duplicates, 158 unique records underwent title and abstract screening; 83 records were excluded, and 75 reports were assessed for full-text eligibility. Six reports were excluded after full-text assessment for the reasons specified in the flow diagram, and 69 database-derived primary studies were retained after final reconciliation. Backward reference and citation screening contributed 15 additional unique eligible primary studies, and one further eligible study was identified during the final literature update, resulting in 85 unique primary studies in the final evidence synthesis.

The database searches identified 526 records: 60 in PubMed, 279 in Scopus, and 187 in Web of Science Core Collection. After removal of 368 duplicate records, 158 unique records underwent title and abstract screening. Eighty-three records were excluded at this stage, and 75 reports were assessed for full-text eligibility. Six reports were excluded at the full-text stage for the reasons specified in Figure 1 and Supplementary Table S1. Following full-text assessment and final reconciliation, 69 database-derived primary studies were retained. Backward reference and citation screening identified 17 potentially eligible primary reports, two of which were already represented in the database-derived corpus; the remaining 15 unique studies were additionally included. A final literature update identified one additional eligible primary study that met the same predefined inclusion criteria. Accordingly, the primary evidence synthesis comprised 85 unique primary studies. The literature identification and study-selection process is summarized in Figure 1, and a study-level inventory of the included primary studies is provided in Supplementary Table S1.

## 2. Introduction

Growing interest in plant-derived bioactive compounds has intensified research into medicinal plant species rich in structurally diverse secondary metabolites with antioxidant, anti-inflammatory and cytotoxic or antiproliferative potential. Secondary metabolites produced by plants, including flavonoids, phenolic acids, terpenoids, and steroidal saponins, are particularly promising because of their structural diversity and broad spectrum of biological activities [2,3]. Despite substantial progress in natural product research, many medicinal plant species, including members of the genus *Dracaena*, remain incompletely characterized with respect to both their phytochemical composition and mechanisms of action.

The genus *Dracaena* (family Asparagaceae) represents one such group of plants with considerable, yet incompletely explored, phytochemical and pharmacological diversity. Comprising numerous species distributed across tropical and subtropical regions of Africa, Asia, and the Middle East, the genus has a documented history of use in traditional medicine. For *D. trifasciata* and *D. angolensis*, ethnobotanical studies report traditional applications involving wounds, infections, inflammatory conditions, snake bites, and other ailments [4,5,6,7]. Ethnopharmacological reports frequently identify leaf, rhizome, and root-derived preparations as major sources of biologically active extracts, supporting continued phytochemical and pharmacological investigation of these species.

Two species stand out as subjects of particular scientific and applied interest. *Dracaena trifasciata* (syn. *Sansevieria trifasciata*) is among the most widely cultivated and commercially important members of the genus; beyond its well-known ornamental value, it has attracted growing pharmacological research focused on metabolic, antimicrobial, anti-inflammatory, and cytotoxic activities [8,9,10,11]. *Dracaena angolensis* (syn. *Sansevieria cylindrica*) has attracted increasing scientific interest because of its distinctive phytochemical profile and emerging pharmacological evidence [12,13,14]. Although it has been investigated less extensively than *D. trifasciata*, its distinctive phytochemical profile suggests considerable potential for further pharmacological investigation. Crucially, both species share a partially overlapping secondary metabolite repertoire—dominated by steroidal saponins, homoisoflavonoids, and phenolic derivatives—yet differ in their specific compound profiles and reported biological activities, making their comparative analysis both scientifically informative and taxonomically meaningful [2,3].

Phytochemical investigations have confirmed the presence of several structurally unique metabolites in these species, including trifasciatosides and related steroidal glycosides [15,16], as well as homoisoflavonoid and dihydrochalcone derivatives identified in aerial parts and rhizomes [12,13,17]. Experimental studies have demonstrated in vitro cytotoxic or antiproliferative effects in selected cancer cell lines and antimicrobial activity against several bacterial species, although the magnitude and reproducibility of these effects vary substantially among preparations and experimental models [11,14,18,19]. Recent advances in chromatographic and metabolomic profiling have further expanded the known chemical inventory of these plants. Mechanistic studies have also begun to associate selected biological effects with NF-κB-related signalling, Nrf2/ARE-associated responses, apoptosis-related markers, and bacterial quorum-sensing regulation; however, the level of experimental support differs substantially among these proposed mechanisms [9,14,20].

Nevertheless, the existing literature on *Dracaena* species has several critical limitations. Studies are largely fragmented across individual biological activities, with few attempts to integrate phytochemical composition with pharmacological and mechanistic evidence. Comparative analyses between *D. trifasciata* and *D. angolensis* are limited, despite the biological and structural relevance of such a comparison. Furthermore, many reported activities lack molecular-level mechanistic characterization, and the compounds responsible for the observed effects have often not been unambiguously identified. Collectively, these gaps limit the translational value of the available data and hinder the rational exploitation of these species as sources of new bioactive leads.

The present review addresses this need by providing a comprehensive and critical synthesis of current knowledge concerning the phytochemical constituents, pharmacological properties and available mechanistic evidence related to *D. trifasciata* and *D. angolensis*. Particular emphasis is placed on the relationship between phytochemical composition, biological activity and intracellular signalling pathways, with special focus on inflammation, oxidative stress, metabolic disorders and cytotoxic/antiproliferative effects. Furthermore, current knowledge gaps and future research directions are discussed in relation to the translational potential of these species as sources of novel bioactive compounds.

## 3. Taxonomy and Botanical Characteristics

The genus *Dracaena* Vand. ex L. (family Asparagaceae) comprises more than 200 accepted species distributed predominantly across tropical and subtropical regions [21]. Historically, many species now classified within *Dracaena* were assigned to the separate genus *Sansevieria* Thunb. However, molecular phylogenetic analyses have demonstrated that *Sansevieria* is nested within *Dracaena*, providing the phylogenetic basis for the inclusion of the former genus within *Dracaena* [22,23]. Current taxonomic databases recognize *Dracaena trifasciata* (Prain) Mabb. and *Dracaena angolensis* (Welw. ex Carrière) Byng & Christenh. as the accepted names, whereas *Sansevieria trifasciata* Prain and *Sansevieria cylindrica* Bojer ex Hook., respectively, are treated as synonyms [21]. Both synonyms remain in common use in the pharmacological and ethnobotanical literature, so both nomenclatures are retained where needed for bibliographic accuracy in this review.

*Dracaena trifasciata* is a perennial succulent with erect, rigid leaves arising from a basal rosette, and it typically shows dark green banding on a lighter background with yellow or white marginal striping. The species exhibits exceptional tolerance to adverse environmental conditions, including low light, irregular watering, and poor substrate quality, which explains its status as one of the most widely cultivated indoor ornamental plants worldwide. Beyond its horticultural importance, *D. trifasciata* produces a well-developed rhizome system that has been identified as a particularly rich source of secondary metabolites, and the species has accordingly attracted sustained phytochemical and pharmacological interest [15,16].

*Dracaena angolensis* is a morphologically distinct species and is widely grown as an ornamental plant. It is a rhizomatous species native to southern tropical Africa and has also attracted scientific attention because of its phytochemical profile and potential medicinal properties [12,13,17,24].

Both species have a long record of traditional medicinal use across multiple regions. Ethnobotanical and ethnopharmacological studies report their use for wounds, infections, inflammatory conditions, snake bites, and other ailments [4,5,6,7]. These traditional uses, together with the documented occurrence of flavonoids, saponins, phenolic compounds, steroids, and terpenoids, have provided a rationale for subsequent phytochemical and pharmacological investigation. This convergence of morphological adaptability, widespread cultivation, and traditional medicinal use provides a compelling rationale for comparative phytochemical and pharmacological investigation of both taxa.

## 4. Ethnomedicinal Uses

*Dracaena trifasciata* has a documented history of use in traditional medicine across geographically and culturally distinct regions, particularly in Africa, Southeast Asia, and Latin America. Primary ethnobotanical studies indicate that the species has been incorporated into local medicinal practices for a range of conditions, although the plant parts used, methods of preparation, and routes of administration vary considerably among communities [4,5,6,7]. These reports provide evidence of traditional use but should not be interpreted as demonstrating pharmacological or clinical efficacy.

In the Mbanza-Ngungu region of the Democratic Republic of Congo, an ethnobotanical survey involving 51 traditional healers documented 195 medicinal plants, including 165 botanically identified species. *Sansevieria trifasciata* was among the species showing the maximum informant agreement ratio (med.IAR = 1), indicating complete agreement among informants who reported its medicinal use [7]. In the survey as a whole, leaves and roots accounted for 63% of cited plant parts, decoction was the most frequent preparation method (46%), and oral administration was the predominant route (71%); however, these aggregate values describe the complete medicinal-plant dataset and should not be attributed specifically to *D. trifasciata*.

Traditional use of *D. trifasciata* has also been documented in Latin America. In an ethnobotanical investigation conducted in five regions of Antioquia, Colombia, Vásquez et al. recorded medicinal plants used by healers and other knowledgeable practitioners for the treatment of snakebite. *S. trifasciata* was among the species documented for this purpose. Across the surveyed medicinal plants, preparations included decoctions and macerations and were administered using several traditional routes [4]. The study documents ethnomedicinal use rather than experimental antivenom efficacy, and these two levels of evidence should therefore be clearly distinguished.

Evidence from East Africa further demonstrates the incorporation of the species into local healing practices. Among Kuria communities, *D. trifasciata* was recorded as a medicinal herb used for the treatment of sores, with the study providing species-specific information on the plant material and topical mode of application [6]. In Southeast Asia, *D. trifasciata* has likewise been documented among medicinal plants used by the Mon people of Myanmar [5].

Compared with *D. trifasciata*, primary ethnobotanical documentation specifically attributable to *D. angolensis* is substantially more limited in the literature identified in the present review. This imbalance parallels the broader disparity in experimental investigation of the two species and highlights the need for better documentation of species-specific traditional uses, plant parts, preparation methods, and routes of administration for *D. angolensis*.

Overall, the available ethnobotanical evidence demonstrates geographically diverse traditional use of *D. trifasciata*, but the reported indications and preparation practices are heterogeneous and remain unevenly documented. Traditional-use reports are therefore considered here as ethnomedicinal evidence and as a basis for generating pharmacological hypotheses, rather than as evidence of therapeutic efficacy.

Importantly, direct experimental validation of the specific preparations and routes of administration described in ethnobotanical studies remains limited. Several categories of traditional indications show broad correspondence with experimentally investigated biological activities; for example, antimicrobial activity has been reported alongside traditional uses related to wounds and infections, while anti-inflammatory and analgesic effects have been investigated in experimental models. However, this correspondence occurs primarily at the level of broad activity categories and should not be interpreted as direct validation of traditional therapeutic claims.

In many cases, the plant preparations used experimentally also differ from those reported in traditional practice: laboratory studies frequently employ ethanolic or methanolic extracts, whereas ethnomedicinal preparations may involve aqueous decoctions, poultices, infusions, or fresh plant material. This preparation gap further limits direct translation between ethnobotanical reports and experimental pharmacological evidence. Studies evaluating traditional preparations under standardized experimental conditions, including direct comparisons of aqueous and organic extracts for the same biological endpoints, would help address this gap. The phytochemical constituents that provide a basis for further experimental investigation of these traditional uses are discussed in the following section.

## 5. Phytochemical Composition of *D. trifasciata* and *D. angolensis*

The genus *Dracaena* is characterized by remarkable diversity in secondary metabolites, and *D. trifasciata* and *D. angolensis* are among the phytochemically investigated herbaceous species within the genus. Extensive investigations show that *D. trifasciata* and *D. angolensis* contain structurally diverse compounds representing several major phytochemical classes, including steroidal saponins, flavonoids, homoisoflavonoids, phenolic acids, and terpenoids [12,13,15,16]. The major phytochemical constituents identified to date are summarized in Table 1.

Detailed characterization of the phytochemical profiles of these species has been made possible by advances in chromatographic and spectrometric techniques. The application of high-performance liquid chromatography (HPLC), liquid chromatography–mass spectrometry (LC-MS), gas chromatography–mass spectrometry (GC-MS) and metabolomic profiling has enabled systematic identification of both polar and non-polar metabolites in extracts derived from leaves, rhizomes and aerial parts [13,20,25,26]. These analyses reveal complex phytochemical profiles; however, the detection of individual metabolites or metabolite classes does not by itself establish their contribution to the biological activity of a crude extract. Compound–activity relationships are therefore considered only where the activity of an isolated compound or chemically characterized fraction was experimentally evaluated.

**Table 1:** Representative phytochemicals identified in *Dracaena trifasciata* and *D. angolensis*, together with plant source, analytical approach, and biological evidence where experimentally evaluated.

Representative Phytochemical(s)	Chemical Class	Species	Plant Part	Isolation/Analytical Approach	Experimentally Reported Biological Relevance	Reference
Trifasciatosides A–J	Steroidal saponins	*D. trifasciata*	Aerial parts	Methanol extract; *n*-BuOH fraction; chromatographic isolation and spectroscopic elucidation	Compound-specific biological activity was not evaluated in this study	[15]
Trifasciatine A and B	Sappanin-type homoisoflavonoids	*D. trifasciata*	Aerial parts	Methanol extract; EtOAc fraction; chromatographic isolation and spectroscopic elucidation	No significant antiproliferative activity against HeLa cells relative to 5-FU	[27]
Trifasciatine C; trifasciatosides K/L and M/N	Dihydrochalcone derivative; steroidal saponins	*D. trifasciata*	Aerial parts	Methanol extract; EtOAc fraction; chromatographic isolation and spectroscopic elucidation	No significant antiproliferative effect was observed for the tested saponin pairs in HeLa cells	[16]
Untargeted metabolomic profile (61 leaf and 59 root metabolites)	Steroidal saponins, phenolics and terpenoids	*D. trifasciata*	Leaves and roots	HPLC-PAD-ESI/MS metabolomic profiling	Leaf and root extracts showed hepatoprotective effects in a TAA rat model and changes in Nrf2/ARE-associated gene-expression markers; the contribution of individual detected metabolites was not established	[20]
Flavonoid-rich extract	Flavonoids	*D. trifasciata*	Leaves	Microwave-assisted enzymatic extraction (MAEE)	MAEE increased extraction yield and flavonoid content; the resulting extract also showed anti-inflammatory activity in an in vivo rat model at 500 mg/kg, although the contribution of individual flavonoids was not established	[28]
Flavonoid-rich extract and solvent fractions	Mixed phytochemical profile	*D. trifasciata*	Leaves	Solvent extraction; GC-MS profiling	In vitro α-glucosidase inhibition was demonstrated; this constitutes enzyme-inhibitory rather than in vivo antidiabetic evidence	[29]
Rutin, kaempferol; total phenolic and flavonoid contents	Flavonoids; phenolic compounds	*D. trifasciata*	Leaves	TPC/TFC assays; HPLC identification	Chemical antioxidant capacity varied among varieties and extraction conditions and was associated with differences in phenolic/flavonoid content	[30]
Representative volatile phytochemicals (including citronellol)	Volatile and semi-volatile metabolites	*D. trifasciata*	Leaves	GC-MS profiling of field- and in vitro raised plants	Differences in chemical profiles were accompanied by differences in in vitro antibacterial activity; causality was not established for individual GC-MS-detected constituents	[25]
2′,4′-Dihydroxy-3′-methoxy-3,4-methylenedioxy-8-hydroxymethylene dihydrochalcone; ruscogenin-type steroidal saponins	Dihydrochalcone derivative; steroidal saponins	*D. angolensis*	Aerial parts	70% aqueous methanol extract; chromatographic isolation and NMR/MS elucidation	Selected isolated compounds showed in vitro cytotoxicity against HCT116, MCF-7 and HepG2 cells; activity varied among compounds	[17]
Representative homoisoflavanones; trifasciatine C; stigmasterol; ergosterol peroxide	Homoisoflavonoids; dihydrochalcone derivative; sterols	*D. angolensis*	Rhizomes	Methanol extract; chromatographic isolation and spectroscopic elucidation	Selected compounds showed weak DPPH radical-scavenging activity; compounds tested in HeLa cells showed no significant cytotoxicity (IC_50_ > 80 µM)	[12]
Representative phenolic constituents (trifasciatine A, cambodianol, hydroxychavicol, *N*-*p*-coumaroyl tyramine derivatives, furanoflavones, 3-methoxyflavone and homopterocarpin)	Phenolic compounds	*D. angolensis*	Rhizomes	Silica gel and reversed-phase chromatographic separation; NMR, CD and HRMS identification	Hydroxychavicol showed the strongest DPPH radical-scavenging activity (EC_50_ = 2.33 μM); selected compounds tested in HeLa cells showed low or no cytotoxicity (IC_50_ > 80 µM)	[13]

Abbreviations: CD, circular dichroism; 5-FU, 5-fluorouracil; DPPH, 2,2-diphenyl-1-picrylhydrazyl; EtOAc, ethyl acetate; GC-MS, gas chromatography–mass spectrometry; HPLC, high-performance liquid chromatography; HPLC-PAD-ESI/MS, high-performance liquid chromatography with photodiode array detection coupled to electrospray ionization mass spectrometry; HRMS, high-resolution mass spectrometry; MAEE, microwave-assisted enzymatic extraction; MS, mass spectrometry; NMR, nuclear magnetic resonance; Nrf2/ARE, nuclear factor erythroid 2-related factor 2/antioxidant response element; TAA, thioacetamide; TFC, total flavonoid content; TPC, total phenolic content; EC_50_, half-maximal effective concentration; IC_50_, half-maximal inhibitory concentration. Note: Only representative phytochemicals and studies directly relevant to their chemical characterization or experimentally evaluated biological activity are included. Detection or identification of a compound in an extract was not considered evidence of its contribution to the biological activity of that extract unless compound-specific activity was experimentally evaluated.

### 5.1. Steroidal Saponins

Steroidal saponins are among the most characteristic secondary metabolites identified in *D. trifasciata* and *D. angolensis* and constitute one of the principal phytochemical groups within the Asparagaceae family. These amphiphilic glycosides consist of a steroidal aglycone linked to one or more sugar residues, and their biological activity is strongly influenced by both the aglycone structure and glycosylation pattern [3]. Phytochemical investigations of *D. trifasciata* and *D. angolensis* have consistently identified steroidal saponins as prominent constituents of their phytochemical profiles. These compounds have been isolated from aerial parts, leaves, and rhizomes in the studies published to date [12,14,15,16,17].

Early phytochemical studies established that the steroidal constituents of *D. trifasciata* are structurally diverse. Steroidal sapogenins, including ruscogenin, neoruscogenin, 25S-ruscogenin and sansevierigenin, were isolated from the leaves, with 25S-ruscogenin and sansevierigenin reported as new spirostan sapogenins [31]. Subsequently, Mimaki et al. [32] isolated 12 steroidal saponins from a methanolic extract of the whole plant, including 10 new constituents, representing the first reported isolation of steroidal saponins from *S. trifasciata*. Steroidal saponins were also reported from the aerial parts of *D. angolensis* in early phytochemical investigations [33], and later isolation studies further expanded this group through identification of structurally characterized steroidal saponins from the species [17,24].

Among the most structurally characterized compounds from *D. trifasciata* are trifasciatosides A-J, a group of diosgenin-type steroidal glycosides differing in their aglycone structures and glycosylation patterns [15]. The key structural features of these compounds are summarized in Table 2. The diversity of glycosylation patterns within this group is particularly noteworthy, as subtle differences in sugar composition and linkage geometry have been shown to exert pronounced effects on biological activity [3]. Structurally related steroidal saponins, although differing in aglycone substitution and sugar chain architecture, have also been reported from *D. angolensis*, further underscoring the phytochemical affinity between the two species [17,24].

The biological relevance of steroidal saponins from the two species remains less clearly established than their phytochemical diversity. Although steroidal saponins as a broader class are known to exhibit membrane-active, cytotoxic, antimicrobial, and anti-inflammatory properties, such general activities should not be attributed directly to the saponins identified in *D. trifasciata* or *D. angolensis* without compound-specific experimental evidence [3]. In *D. trifasciata*, the steroidal saponin pairs trifasciatosides K/L and M/N did not show significant antiproliferative activity against HeLa cells [16]. In *D. angolensis*, individual steroidal saponins isolated from aerial parts showed variable in vitro cytotoxicity against HCT116, MCF-7, and HepG2 cells, indicating that biological activity differs substantially among structurally related compounds [17]. More recently, a higher total steroidal saponin content in rhizomes than in leaves of *D. angolensis* was observed alongside stronger cytotoxic activity of the rhizome extract; however, this association does not establish steroidal saponins as the compounds responsible for the observed effect [14]. Accordingly, mechanistic effects reported for steroidal saponins as a broader phytochemical class should not be regarded as experimentally demonstrated mechanisms of the specific saponins identified in these two species [3].

From a biosynthetic and ecological perspective, steroidal saponins in Asparagaceae are considered constitutive chemical defence compounds, and their membrane-disrupting and bitter-tasting properties help deter herbivores and phytopathogens [3]. This defensive role may explain their particularly high abundance in rhizomes and other storage organs, which are especially vulnerable to soil-dwelling pathogens and invertebrate predation. However, the ecological functions of individual steroidal saponins identified in *D. trifasciata* and *D. angolensis* have not been directly investigated and remain to be established.

### 5.2. Homoisoflavonoids

Homoisoflavonoids are a structurally distinct class of phenolic natural products that are relatively rare in the plant kingdom and occur predominantly in selected monocotyledonous families, including Asparagaceae. They differ from classical flavonoids by the presence of an additional methylene group between the B-ring and the carbonyl-bearing C-ring, resulting in a 3-benzylchroman-4-one (homoisoflavanone) scaffold or related structural variants rather than the typical C6-C3-C6 flavonoid framework. Within Asparagaceae, the occurrence of homoisoflavonoids is considered chemotaxonomically informative, and their distribution across Dracaena, Sansevieria, and related genera has been comprehensively documented [2].

For *D. angolensis* (syn. *S. cylindrica*), the most detailed phytochemical data come from two complementary studies of rhizome material collected in Myanmar. Aye et al. [12] isolated a new sappanin-type homoisoflavanone, (3S)-3-(4-methoxybenzyl)-3,5-dihydroxy-7-methoxy-6-methylchroman-4-one, together with four known congeners: (3S)-3-(4-methoxybenzyl)-3,5-dihydroxy-7-methoxychroman-4-one, (3S)-3-(4-hydroxybenzyl)-3,5-dihydroxy-7-methoxy-6-methylchroman-4-one, (3S)-3-(4-hydroxybenzyl)-3,5-dihydroxy-7-methoxychroman-4-one, and 3-(3,4-methylenedioxybenzyl)-7-hydroxy-8-methoxychroman-4-one. The same study also reported the first natural isolation of the (−)-enantiomer of trifasciatine C. Structural elucidation was performed using extensive 1D and 2D NMR spectroscopy together with mass spectrometry.

Subsequently, Aung et al. [13] isolated ten phenolic compounds representing five different chemical classes from *S. cylindrica* rhizomes. Several of these compounds, including additional homoisoflavanones, were reported for the first time from a *Sansevieria* species, while five compounds had not previously been described within the Asparagaceae family.

For *D. trifasciata*, reports on homoisoflavonoid isolation remain more limited than those on steroidal saponins. Nevertheless, Tchegnitegni et al. [16] described the isolation of trifasciatine C, a dihydrochalcone derivative structurally related to the homoisoflavanone series, from the aerial parts of *S. trifasciata*, alongside several novel steroidal saponins. Metabolomic studies have further supported the presence of diverse phenolic constituents in *D. trifasciata* extracts, although compound-level characterization of homoisoflavonoids remains considerably less advanced than that reported for *D. angolensis* rhizomes [2,20].

The currently available evidence indicates that homoisoflavonoids and related phenolic compounds isolated from *D. angolensis* rhizomes generally show limited cytotoxicity, whereas their antioxidant activity varies among individual compounds. Homoisoflavanones isolated from *D. angolensis* rhizomes showed no significant cytotoxic activity against HeLa cells, whereas most of the individual compounds tested showed weak activity in the 2,2-diphenyl-1-picrylhydrazyl (DPPH) radical-scavenging assay [12]. Aung et al. [13] similarly reported low or no cytotoxicity for selected compounds tested against HeLa cells, although hydroxychavicol showed comparatively strong DPPH radical-scavenging activity (EC_50_ = 2.33 µM). This pattern—variable antioxidant activity coupled with generally limited cytotoxicity-is broadly consistent with findings reported for homoisoflavonoids from other Asparagaceae genera, in which antioxidant and anti-inflammatory activities have also been described [2].

Studies of homoisoflavonoids from other plant taxa have suggested that inhibition of NF-κB activation, together with reduced expression of COX-2 and iNOS, may contribute to their anti-inflammatory activity [2]. However, these mechanisms have not been directly demonstrated for the specific homoisoflavonoids isolated from *D. trifasciata* or *D. angolensis* and therefore remain mechanistic hypotheses requiring experimental validation. Collectively, the homoisoflavonoid constituents isolated from *D. angolensis* rhizomes represent one of the most comprehensively characterized groups of individual metabolites reported for herbaceous *Dracaena* species. In contrast, the homoisoflavonoid profile of *D. trifasciata* remains incompletely characterized and warrants further isolation-guided phytochemical and biological investigation.

### 5.3. Flavonoids

Flavonoids are among the most consistently reported polyphenolic metabolites identified in *D. trifasciata* and, to a lesser extent, in *D. angolensis*. Within the *Dracaena* and *Sansevieria* genera, flavonoids and stilbenoids together form a major class of pharmacologically relevant secondary metabolites, and their structures and biological activities have been comprehensively reviewed by Thu et al. [2].

Preliminary phytochemical screening studies consistently reported the presence of flavonoids in different cultivars and solvent extracts of *D. trifasciata*, providing an early basis for subsequent compound-level characterization [34]. Fawaz et al. [35] isolated and identified several phenolic constituents from the ethyl acetate fraction of *S. trifasciata* leaves, including the flavonoids kaempferol, quercetin and catechin, together with the phenolic acid chlorogenic acid. These compounds were identified using HPLC and spectroscopic techniques, and the authors reported their isolation from this species for the first time. Among the identified metabolites, kaempferol and catechin were the predominant constituents.

A subsequent metabolomic investigation by Raslan et al. [20] using high-performance liquid chromatography coupled with photodiode array detection and electrospray ionization mass spectrometry (HPLC-PAD-ESI/MS) tentatively identified 61 compounds in leaf extracts and 59 compounds in root extracts of *S. trifasciata*. Phenolic compounds, together with steroidal saponins and terpenoids, constituted a substantial proportion of the phytochemical profile.

More recently, Saputra et al. [30] evaluated four cultivars of *D. trifasciata* and identified rutin and kaempferol in ethanolic leaf extracts by HPLC analysis. Total flavonoid content varied markedly among cultivars, with the highest value (368.96 mg QE/g extract) observed in the ‘Gold Flame’ cultivar. The authors further demonstrated a strong positive correlation between total flavonoid content and antioxidant activity, as measured by the DPPH, CUPRAC, and FRAP assays. Despite differences in extraction procedures, cultivars and analytical methods, these findings support an association between flavonoid abundance and chemical antioxidant capacity, although they do not establish the contribution of individual flavonoids to the observed activity.

Additional evidence for the biological relevance of flavonoid-containing extracts was provided by Yumna et al. [29], who demonstrated that polar leaf extracts containing flavonoid constituents inhibited α-glucosidase activity in vitro. However, because individual flavonoids were not evaluated separately, their specific contribution to the observed enzyme-inhibitory activity remains unresolved.

For *D. angolensis*, compound-level information on flavonoids remains limited. Phytochemical screening of leaf extracts confirmed the presence of flavonoids and tannins, while the methanolic leaf fraction showed pronounced DPPH radical-scavenging activity (83.6% DPPH inhibition at 100 μg/mL) and inhibited glucose-6-phosphatase activity by 57.9% [36]. These findings demonstrate chemical antioxidant capacity and enzyme-inhibitory activity of the fraction, but do not establish flavonoids as the compounds responsible for these effects. However, no studies retrieved in the present review reported the isolation and structural characterization of individual flavonoids from *D. angolensis*, highlighting a significant gap in the current phytochemical knowledge of this species.

Beyond their antioxidant properties, flavonoids identified in *Dracaena* species may also contribute to anti-inflammatory and cytoprotective activities through modulation of intracellular signalling pathways, particularly NF-κB and Nrf2/ARE. In the study by Raslan et al. [20], the hepatoprotective effects of *S. trifasciata* extracts in a thioacetamide-induced liver fibrosis model were associated with changes in Nrf2/ARE-related markers and increased expression of antioxidant defence markers, including glutathione (GSH), heme oxygenase-1 (HO-1), and NAD(P)H quinone oxidoreductase-1 (NQO-1). Nevertheless, the specific contribution of individual flavonoids to these effects remains unresolved.

Collectively, the available evidence indicates that flavonoids are prominent constituents of the phytochemical profile of *D. trifasciata*, with kaempferol, quercetin, rutin, and catechin representing the best-characterized individual compounds identified to date. In contrast, flavonoids in *D. angolensis* remain poorly characterized at the molecular level, and systematic, isolation-guided studies are needed to clarify their structures and biological activities and to determine their potential pharmacological relevance.

### 5.4. Phenolic Acids and Polyphenols

Phenolic acids and other low-molecular-weight phenolic compounds represent a major fraction of the water- and alcohol-soluble metabolites in both *D. trifasciata* and *D. angolensis*, and their abundance is frequently associated with chemical antioxidant capacity across studies using different extraction and quantification methods. The most detailed compound-level characterization comes from *D. trifasciata*, where HPLC analysis of ethyl acetate fractions enabled the isolation and spectroscopic identification of the phenolic acid chlorogenic acid together with the flavonoids quercetin, kaempferol and catechin from the leaves, as well as apigenin, luteolin, caffeic acid and chlorogenic acid from the roots, all reported for the first time from this species [37]. An earlier HPLC-based comparison of two *Sansevieria* species by Fawaz et al. [35] confirmed the presence of chlorogenic acid, quercetin, kaempferol, and catechin in the leaf ethyl acetate fractions of *S. trifasciata*, with catechin and kaempferol identified as the major compounds; importantly, *p*-hydroxybenzoic and *p*-coumaric acids were detected in the related species *S. suffruticosa* but not in *S. trifasciata*, suggesting species-level variation in hydroxycinnamic acid composition.

The most comprehensive profile of the phenolic metabolome in *D. trifasciata* was reported by Raslan et al. [20], who used HPLC-PAD-ESI/MS to tentatively identify 61 compounds in leaf extracts and 59 in root extracts, with phenolic compounds representing one of the principal phytochemical groups alongside steroidal saponins and terpenoids.

GC-MS analysis of the ethanolic leaf extract additionally tentatively identified 31 volatile and semi-volatile compounds, with phenolic-type compounds, including Phenol, 4,4′-thiobis[2,6-dimethyl-], among the major annotated constituents [38]. While GC-MS captures a different chemical window than HPLC-based methods, the available data collectively suggest that the phenolic profile of *D. trifasciata* is chemically diverse and extraction-method dependent, which is relevant for standardizing any derived preparation.

For *D. angolensis*, compound-level identification of individual phenolic acids remains limited. Phytochemical screening of *S. cylindrica* leaf fractions confirmed the presence of phenolic acids, flavonoids, tannins, and saponins, with the methanol fraction showing the highest phenolic content [36]. In contrast, GC-MS analysis predominantly identified sterols and fatty acid esters, whereas phenolic compounds represented only a minor fraction of the detected metabolites [14]. Although several individual phenolic constituents have been isolated from *D. angolensis* rhizomes [12,13], compound-level characterization of phenolic acids remains substantially less developed than in *D. trifasciata*.

The currently available evidence for phenolic acids and related polyphenols is uneven between the two species. *D. trifasciata* has a reasonably defined phenolic profile at the compound level, with chlorogenic and caffeic acids representing the best-characterized phenolic acids, alongside the flavonoids discussed in Section 5.3. In contrast, compound-level characterization specifically of phenolic acids in *D. angolensis* remains substantially more limited, despite the isolation of other phenolic constituents from rhizome material. Systematic isolation-guided studies of *D. angolensis* tissues, particularly the rhizomes, are needed to determine whether the two species share overlapping phenolic acid profiles or are chemically distinct.

### 5.5. Terpenoids and Triterpenes

Terpenoids constitute a chemically diverse class of secondary metabolites that have been consistently detected in extracts of both *D. trifasciata* and *D. angolensis*. Compared with steroidal saponins and flavonoids, however, compound-level characterization of individual terpenoids remains considerably less advanced. Primary phytochemical studies have identified terpenoids, sterols and triterpenoid-type compounds in leaves and roots of both species, although the extent of compound-level characterization varies among studies. Phytochemical screening of ethanol leaf extracts from both *S. trifasciata* and *S. cylindrica* further confirmed the presence of triterpenoid and steroidal compounds in both species, with *S. trifasciata* additionally showing flavonoids and *S. cylindrica* showing alkaloids as co-dominant screening-positive classes [39].

Despite their consistent detection in phytochemical screening studies, only a limited number of individual terpenoid constituents have been characterized at the compound level. Among these, volatile diterpenoids detected by GC-MS have received the greatest attention. GC-MS analysis of antibacterially active *D. trifasciata* leaf fractions identified neophytadiene as a major constituent of an active fraction; however, its individual contribution to the antibacterial activity was not experimentally established [40]. GC-MS profiling of *S. trifasciata* leaves additionally revealed isophytol and a range of alkanes, esters, and terpenoid-type constituents among the reported phytochemicals [26].

For *D. angolensis*, the rhizome GC-MS study by Ibrahim et al. [14] identified beta-sitosterol as the predominant GC-detectable compound, accounting for 16.13% of the rhizome extract, while fatty acids and their esters constituted most of the remaining volatile profile.

Pentacyclic triterpenes have been reported from other *Dracaena* species; however, primary isolation-level evidence for lupeol, ursolic acid, betulinic acid or related pentacyclic triterpenes from *D. trifasciata* or *D. angolensis* was not identified in the literature retrieved for this review. Their occurrence and biological relevance in the two target species therefore remain to be established.

Overall, the available evidence documents phytosterol and volatile diterpenoid-related constituents in the two species, whereas the non-saponin triterpene fraction remains insufficiently characterized.

### 5.6. Pregnane, Furostanol and Related Steroidal Glycosides

Beyond the steroidal saponins discussed in Section 5.1, phytochemical investigations of *D. trifasciata* and *D. angolensis* have identified additional classes of steroidal glycosides, including pregnane and furostanol glycosides. Closely related *Dracaena* species have also yielded structurally distinct C-22 steroidal lactone glycosides, known as dracaenosides. Together, these compounds considerably broaden the structural diversity of steroidal glycosides within the genus and provide a useful framework for interpreting biological activities that may not be explained by saponin content alone.

The most structurally characterized non-saponin glycosides from *D. trifasciata* are the pregnane glycosides first isolated from the whole plant by Mimaki et al. [41]. Four new pregnane glycosides were identified; spectroscopic analysis and hydrolysis established them as 1β,3β-dihydroxypregna-5,16-dien-20-one glycosides, a structural class not previously reported from any Agavaceae plant at that time. Subsequently, Tchegnitegni et al. [16] isolated trifasciatosides K/L and M/N, four previously unreported steroidal saponins obtained as two pairs of inseparable regioisomers, together with the new dihydrochalcone derivative trifasciatine C from the aerial parts of *S. trifasciata*. The isolated regioisomeric pairs K/L and M/N showed no significant antiproliferative activity against HeLa cells.

For *D. angolensis* (syn. *S. cylindrica*), Da Silva Antunes et al. [42] reported the isolation of a new furostanol glycoside from the leaves. The sugar chain consisted of two mannose residues and one glucose unit, and the furostanol core bore hydroxyl substituents at C-12 and C-15, a substitution pattern unusual within the genus. The compound exhibited no haemolytic activity and reduced capillary permeability, demonstrating biological activity in the experimental assay, although a specific anti-inflammatory mechanism was not established. Phytochemical screening of methanolic extracts from both the leaves and rhizomes of *S. cylindrica* confirmed the presence of steroidal saponins; notably, the rhizome extract contained approximately twice the saponin content of the leaf extract (6.65 vs. 3.88 mg/g), although the study did not provide compound-level characterization of individual glycosides [14].

The term dracaenoside, in the stricter compound-level sense, refers to C-22 steroidal lactone glycosides first isolated from *Dracaena cochinchinensis* by Zheng et al. [43], where dracaenosides A and B were characterized as C-22 steroidal lactone glycosides bearing rhamnose- and glucose-containing oligosaccharide chains, representing the first steroidal lactone glycosides isolated from the genus. This structural class has not, to the authors’ knowledge, been isolated specifically from *D. trifasciata* or *D. angolensis*; in secondary literature, the term is sometimes used more loosely to refer to steroidal glycosides from *Dracaena* sensu lato, which can lead to terminological confusion.

### 5.7. Other Phytochemical Constituents

In addition to the major classes of secondary metabolites discussed above, phytochemical studies have identified a range of minor constituents in *D. trifasciata* and *D. angolensis*, including chlorophyll pigments, carotenoids, volatile and lipophilic metabolites, fatty acids, and selected nutritional components. Although these compounds are consistently detected by chromatographic and spectroscopic analyses, they have received considerably less attention than steroidal saponins, flavonoids, or phenolic acids, and their contribution to the biological activities of crude extracts remains poorly understood.

Available information on photosynthetic pigments is limited. For *D. trifasciata*, chlorophyll variation has been evaluated mainly at the cultivar level, with cv. *African Dawn* exhibiting the highest chlorophyll content among the cultivars examined [44]. However, no studies have directly investigated whether chlorophylls or carotenoids contribute independently to the antioxidant properties of *D. trifasciata* or *D. angolensis* extracts.

The lipophilic fraction has been characterized more extensively using GC-MS. GC-MS analysis of ethanolic extracts revealed numerous putatively identified volatile and semi-volatile constituents, including diterpenoid-related compounds, fatty acids, fatty acid esters, hydrocarbons, sterols, and phenolic-type compounds [25,26,38,45]. In the unsaponifiable fraction of the aerial non-flowering parts of D. trifasciata, phytol was the predominant GC-MS-detected constituent (48.15%), followed by γ-sitosterol (13.39%), stigmasterol (11.01%) and β-sitosterol (10.55%) [45]. Among the repeatedly reported constituents are neophytadiene, isophytol, palmitic acid, methyl linoleate, methyl linolenate, linoleic acid, oleic acid, and stearic acid [25,26,38]. Collectively, these findings indicate that the lipophilic fraction of *D. trifasciata* is dominated by long-chain fatty acids, their esters, diterpenoid-derived compounds, and other non-volatile lipophilic metabolites rather than by classical monoterpene-rich essential oils. Accordingly, *D. trifasciata* should not be regarded as a typical essential-oil-producing medicinal plant on the basis of the currently available compositional evidence.

Among nutritional constituents, vitamin E has been quantified in ethanolic leaf extracts of *D. trifasciata* (41.24 μg/mL) [38]. Comparable vitamin analyses are currently unavailable for *D. angolensis*.

Overall, the available evidence confirms the presence of pigments, lipophilic metabolites, fatty acids, and selected nutritional constituents in *D. trifasciata* and, to a much lesser extent, *D. angolensis*. However, current knowledge remains largely descriptive, and few studies have evaluated the independent biological significance of these minor phytochemical constituents. Future investigations integrating comprehensive GC-MS and LC-MS/MS profiling with bioactivity-guided fractionation will be necessary to determine whether these compounds contribute directly to the pharmacological properties of the crude extracts.

### 5.8. Metabolomic and Chromatographic Profiling of Dracaena Species

The application of advanced chromatographic and metabolomic techniques has substantially improved the phytochemical characterization of *D. trifasciata* and, to a lesser extent, *D. angolensis*. Compared with conventional phytochemical screening, hyphenated analytical platforms have enabled tentative or confirmed identification of individual metabolites, facilitated comparison of different plant organs and extraction procedures, and provided a more comprehensive understanding of the chemical complexity underlying the biological activities of these species.

The most comprehensive metabolomic investigation available for *D. trifasciata* is the HPLC-PAD-ESI/MS study by Raslan et al. [20], which tentatively identified 61 compounds in leaf extracts and 59 compounds in root extracts. The identified metabolites were dominated by phenolic compounds, steroidal saponins and terpenoids, illustrating the broad chemical diversity of both organs and demonstrating the utility of untargeted metabolomic profiling for comprehensive phytochemical characterization. In contrast, the targeted HPLC approach applied by Fawaz et al. [35] focused on confirmation of individual phenolic constituents using authentic standards, illustrating the complementary role of targeted chromatographic analysis in validating metabolites identified through broader phytochemical screening.

GC-MS has primarily been employed to characterize the volatile and lipophilic fractions of *D. trifasciata*. GC-MS profiling revealed putatively identified diterpenoid-derived compounds, long-chain fatty acids, fatty acid esters and other lipophilic constituents, complementing the predominantly polar metabolite profiles obtained by HPLC-based methods [26,38,40]. Particularly noteworthy, Kaur et al. [25] demonstrated that tissue-culture propagation under modified growth conditions substantially altered the GC-MS metabolite profile compared with field-grown plants, indicating that cultivation conditions can significantly influence phytochemical composition. These findings emphasize the importance of standardized cultivation and extraction protocols when comparing pharmacological studies.

For *D. angolensis*, compound-level chromatographic characterization remains considerably less extensive. The most detailed phytochemical investigation is the isolation-guided study of Aung et al. [13], in which repeated silica-gel and reversed-phase chromatographic separations enabled structural elucidation of ten phenolic compounds from rhizome extracts. Together with the work of Aye et al. [12], these studies demonstrate that isolation-guided chromatography can achieve compound-level resolution of rhizome metabolites despite the comparatively limited phytochemical exploration of this species. In contrast, comparative fractionation studies based on VLC and TLC have primarily been used to associate chemically distinct fractions with antibacterial activity rather than to comprehensively characterize individual metabolites [46].

Collectively, the available evidence demonstrates that HPLC- and LC-MS-based techniques are particularly effective for characterizing polar metabolites, including phenolic compounds, flavonoids and steroidal glycosides, whereas GC-MS predominantly resolves volatile and lipophilic constituents. These complementary analytical approaches provide a substantially more comprehensive view of the phytochemical composition than conventional screening methods alone. Nevertheless, untargeted metabolomic profiling comparable to that reported for *D. trifasciata* has not yet been performed for *D. angolensis*, representing an important methodological gap. Future studies integrating high-resolution metabolomics with bioactivity-guided fractionation and quantitative marker validation across comparable plant organs and extraction procedures will be essential for improving cross-species phytochemical comparisons and strengthening structure–activity relationships.

## 6. Pharmacological Activities of *D. trifasciata* and *D. angolensis*

The pharmacological and biological activities of *D. trifasciata* and *D. angolensis* have been increasingly investigated over the past decade, with studies reporting chemical antioxidant capacity, anti-inflammatory and antimicrobial effects, enzyme-inhibitory activity, in vitro cytotoxic and antiproliferative effects, and selected metabolic effects in animal models. These biological effects have been investigated in preparations containing steroidal saponins, flavonoids, phenolic acids, homoisoflavonoids and terpenoids; however, the compounds responsible for the observed activities have not been established in most studies, and differences in metabolite composition between species, plant organs and preparations may contribute to variations in biological activity. A comprehensive summary of the reported biological activities, experimental models, doses or concentrations, comparators, quantitative outcomes, evidence levels, and mechanism status is presented in Table 3.

**Figure 2:**
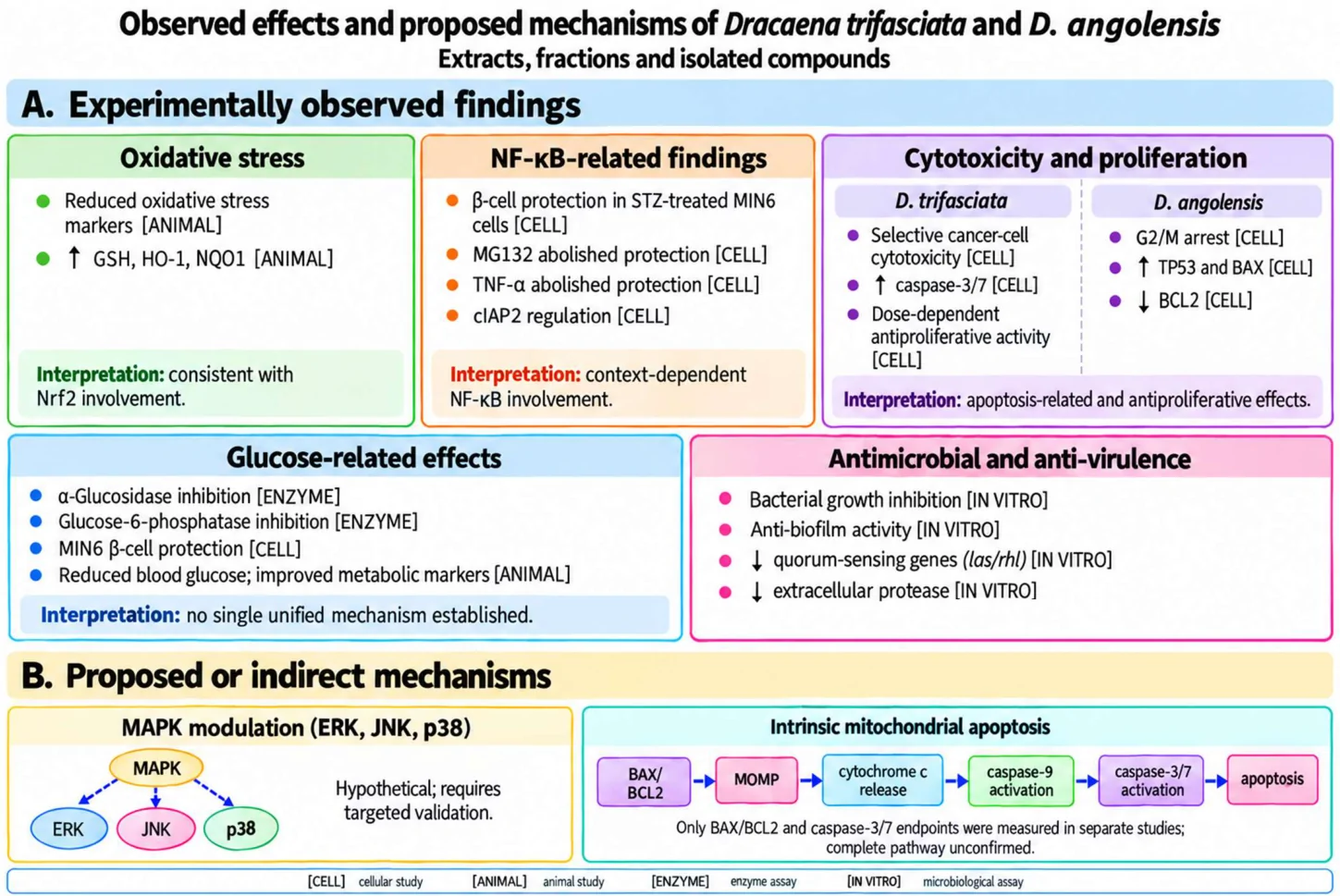
Schematic overview of experimentally observed effects and proposed molecular mechanisms associated with *Dracaena trifasciata* and *D. angolensis*. Experimentally observed findings include Nrf2/ARE-associated responses, context-dependent NF-κB-related effects, apoptosis- and cell-cycle-related changes, glucose-related effects, and antimicrobial and virulence-associated responses. Proposed or indirect mechanisms, including MAPK-related signalling and the complete intrinsic mitochondrial apoptotic pathway, are shown separately to distinguish experimentally observed relationships from hypothesis-generating interpretations. Dashed arrows indicate hypothetical relationships, whereas solid arrows indicate pathway relationships depicted in the proposed mechanistic scheme. Abbreviations: BAX, BCL2-associated X protein; BCL2, B-cell lymphoma 2; ERK, extracellular signal-regulated kinase; GSH, glutathione; HO-1, heme oxygenase-1; JNK, c-Jun N-terminal kinase; MAPK, mitogen-activated protein kinase; MOMP, mitochondrial outer membrane permeabilization; NF-κB, nuclear factor kappa B; NQO1, NAD(P)H quinone oxidoreductase 1; Nrf2, nuclear factor erythroid 2-related factor 2; STZ, streptozotocin; TNF-α, tumour necrosis factor alpha; cIAP2, cellular inhibitor of apoptosis protein 2; TP53, tumour protein p53; MG132, proteasome inhibitor MG132; MIN6, mouse insulinoma 6 cells; p38, p38 mitogen-activated protein kinase.

The experimentally observed effects and proposed molecular mechanisms associated with these pharmacological activities are summarized in Figure 2.

Overall, *D. trifasciata* is considerably better characterized than *D. angolensis* and currently provides the strongest experimental evidence for antioxidant, anti-inflammatory, cytoprotective, metabolic, enzyme-inhibitory, and in vitro cytotoxic and antiproliferative activities. In contrast, pharmacological studies on *D. angolensis* remain relatively limited but indicate a broadly comparable spectrum of biological effects, particularly with respect to antioxidant, antimicrobial, and enzyme-inhibitory activities, together with more limited cytotoxic and other pharmacological evidence.

Despite the growing body of evidence, most studies have evaluated crude or partially purified extracts, limiting the identification of the metabolites responsible for the observed biological activities. Consequently, further bioactivity-guided fractionation, comprehensive phytochemical characterization and mechanistic investigations are required to establish clear compound-activity relationships and to determine whether the observed preclinical activities can support further therapeutic development.

### 6.1. Antioxidant Activity

Antioxidant capacity is among the most consistently documented biological properties of *D. trifasciata* and *D. angolensis*, having been demonstrated using complementary in vitro assays, including the 2,2-diphenyl-1-picrylhydrazyl (DPPH), 2,2′-azinobis-(3-ethylbenzothiazoline-6-sulfonic acid) (ABTS), ferric reducing antioxidant power (FRAP), cupric ion reducing antioxidant capacity (CUPRAC) and phosphomolybdenum assays. Across the available studies, chemical antioxidant capacity is frequently associated with the phenolic and flavonoid content of the extracts, although considerable variability is observed depending on extraction procedure, solvent polarity, plant material and cultivar [30,34,58,62,63,64,65]. Comparison of conventional decoction with microwave-assisted aqueous extraction further demonstrated that extraction methodology markedly influences both total phenolic content and total antioxidant activity, with microwave-assisted extraction producing higher values than the traditional decoction method [62]. Consistent with these observations, ethanolic extracts generally possess higher total phenolic contents and stronger reducing capacity than aqueous extracts, whereas aqueous extracts may exhibit superior DPPH radical-scavenging activity, indicating that extraction conditions influence the recovery of different classes of antioxidant constituents [63]. Further evidence of extraction-dependent variability was provided by a study comparing sequentially obtained chloroform and ethanolic leaf extracts of *D. trifasciata*. Both showed DPPH radical-scavenging activity, with IC_50_ values of 370.8 ± 0.07 and 647.4 ± 0.12 µg/mL, respectively, compared with 4.17 ± 0.02 µg/mL for ascorbic acid [58]. Likewise, ethanolic leaf extracts displayed DPPH radical-scavenging activity together with membrane-stabilizing effects; however, the specific constituents responsible for either effect were not established [52]. More recent extract-level evidence also demonstrated antioxidant activity of ethanolic and methanolic *D. trifasciata* extracts; these extracts were subsequently incorporated into hydrogel formulations [66].

The antioxidant potential of *D. trifasciata* also appears to vary considerably among cultivars. Comparative studies demonstrated significant differences in DPPH, FRAP and CUPRAC activities, which were associated with differences in total phenolic and flavonoid contents. HPLC analyses further identified rutin and kaempferol among the detected flavonoid constituents; however, their individual contribution to the antioxidant activity was not directly established [30]. Similarly, comparative evaluation of several *Sansevieria* species identified *D. trifasciata* as the species with the highest DPPH radical-scavenging activity in the experimental conditions used [64].

Comparable chemical antioxidant capacity has also been reported for *D. angolensis*, whose methanolic leaf fraction exhibited 83.6% DPPH radical-scavenging activity at 100 μg/mL together with high phenolic content [36]. The same fraction inhibited glucose-6-phosphatase activity by 57.9%; however, these parallel findings do not establish phenolic constituents as the compounds responsible for either effect or demonstrate a shared biological mechanism.

Beyond direct free-radical scavenging, experimental evidence indicates that *D. trifasciata* may also influence endogenous antioxidant defence mechanisms. In a thioacetamide-induced liver fibrosis model, treatment with leaf and root extracts was associated with reduced oxidative stress and increased hepatic expression of Nrf2, HO-1, NQO1 and Keap1, together with increased GSH levels [20]. These findings are consistent with modulation of Nrf2/ARE-associated antioxidant responses, although direct pathway activation was not demonstrated.

Collectively, the available evidence identifies chemical antioxidant capacity as one of the most consistently reported biological properties of both *D. trifasciata* and *D. angolensis*. Although the magnitude of the response varies with extraction procedure and plant genotype, the frequent association with phenolic and flavonoid abundance, together with in vivo evidence of changes in endogenous antioxidant defence markers in *D. trifasciata*, supports further investigation of oxidative-stress modulation as a potential contributor to the biological effects of these species.

### 6.2. Anti-Inflammatory Activity

Experimental studies indicate that *D. trifasciata* possesses anti-inflammatory activity in both in vitro and in vivo models, although the molecular mechanisms underlying these effects remain only partially understood. Current evidence suggests that the reported activity may reflect a broader immunomodulatory profile rather than simple suppression of inflammatory responses.

Initial evidence was obtained from membrane stabilization assays, in which ethanolic leaf extracts inhibited hypotonicity- and heat-induced haemolysis, indicating the ability to stabilize cellular membranes and thereby supporting their potential anti-inflammatory activity [52]. Consistent with these findings, in vivo studies demonstrated significant inhibition of egg albumin-induced paw edema in mice following both oral and intraperitoneal administration of ethanolic leaf extracts, demonstrating an anti-inflammatory effect in this animal model [67]. Phytochemical analysis of the active extracts identified terpenes, alkaloids, cardiac glycosides and tannins; however, the contribution of individual phytochemical classes to the observed effect was not established.

Additional support for the cytoprotective potential of *D. trifasciata* was obtained in a zebrafish (*Danio rerio*) model, where aqueous leaf extracts prevented diclofenac-induced mortality. Because this protection was accompanied by attenuation of oxidative stress, the findings suggest a protective effect against diclofenac-induced toxicity, although they do not directly demonstrate an anti-inflammatory mechanism [68].

Mechanistic evidence remains limited but includes evidence of NF-κB involvement in the cytoprotective response described in pancreatic β-cells. *D. trifasciata* leaf extracts protected streptozotocin (STZ)-treated MIN6 pancreatic β-cells, while pharmacological interference with NF-κB-related signalling abolished this cytoprotective effect [9]. Interestingly, the same extract enhanced TNF-α expression in LPS-stimulated macrophages, indicating that its effects on inflammatory signalling are highly context-dependent. These findings support the involvement of NF-κB-related signalling but do not establish direct pathway activation or inhibition by specific extract constituents.

Additional in vitro evidence for the anti-inflammatory potential of *D. angolensis* was obtained using protein denaturation and membrane-stabilization assays, in which hydroalcoholic leaf extracts showed dose-dependent activity together with antioxidant activity [54].

Overall, the available evidence supports the anti-inflammatory activity of *D. trifasciata* and, to a lesser extent, *D. angolensis*, while also indicating that the pharmacological effects of *D. trifasciata* extend beyond classical anti-inflammatory mechanisms. Further studies are required to clarify the molecular targets of individual phytochemicals and to determine how context-dependent regulation of inflammatory signalling contributes to the observed biological effects.

### 6.3. Antimicrobial Activity

Antimicrobial activity is among the best-characterized biological properties of *D. trifasciata*, with consistent evidence demonstrating activity against both Gram-positive and Gram-negative bacteria. Current findings indicate that its antibacterial effects involve not only inhibition of bacterial growth but also interference with virulence-associated processes, including biofilm formation and quorum sensing. In contrast, antimicrobial studies on *D. angolensis* remain comparatively limited.

Multiple studies have demonstrated antibacterial activity of *D. trifasciata* leaf extracts against clinically relevant pathogens. Activity against *Staphylococcus aureus* has been reported in several studies [38,45,57,58,69], while activity against *Escherichia coli* has likewise been documented [38,45,57,69,70,71]. Additional antimicrobial screening studies further support the antibacterial potential of *D. trifasciata*, although the tested microorganisms, extract preparations, and experimental conditions varied among studies [72,73,74]. Additional activity against *Salmonella* spp. and *Bacillus* spp. was reported by Udo et al. [38], although the magnitude of activity varied depending on extraction method, solvent and bacterial strain. Comparative analyses further demonstrated that *D. trifasciata* exhibits stronger antibacterial activity than *D. angolensis* against *P. aeruginosa*. Bioactivity-guided fractionation identified an active fraction containing flavonoids, phenolics, terpenoids and triterpenoids; however, the contribution of individual phytochemical classes to the observed antibacterial activity was not established [46]. Additional studies have reported antibacterial activity of saponin-containing fractions, although the contribution of individual saponins remains to be established [75].

Bioactivity-guided fractionation has also led to the isolation of an antibacterial pyridone alkaloid derivative, 5-methyl-11-(2-oxopyridin-1(2H)-yl)undecaneperoxoic acid, from *D. trifasciata*. The isolated compound exhibited low MIC values against the bacterial strains tested, while molecular docking and MM-PBSA analyses predicted interactions with β-ketoacyl-ACP synthase and tyrosyl-tRNA synthetase [76].

Beyond direct antibacterial activity, recent investigations indicate that *D. trifasciata* also interferes with bacterial virulence mechanisms. Active fractions suppressed *P. aeruginosa* biofilm formation [40] and reduced extracellular protease production together with expression of the *las* and *rhl* quorum-sensing regulatory genes [55]. These findings provide experimental evidence of effects on biofilm formation and quorum-sensing-associated virulence regulation, although the responsible individual metabolites have not yet been established. Such anti-virulence effects may represent an additional direction for antimicrobial investigation beyond direct growth inhibition.

Compared with *D. trifasciata*, the antimicrobial properties of *D. angolensis* remain less comprehensively investigated. Available studies demonstrate antibacterial activity of *D. angolensis* leaf extracts against *E. coli*, *S. aureus* and *P. aeruginosa* [19,59]. Methanolic leaf and rhizome extracts have additionally shown activity against *Agrobacterium tumefaciens* and *Serratia marcescens*, as well as antifungal activity against *Fusarium oxysporum* [14]. However, the metabolites responsible for these activities have not yet been identified, and the underlying molecular mechanisms remain largely unknown.

Current evidence therefore identifies *D. trifasciata* as the better-characterized antimicrobial species, with experimental support for bacterial growth inhibition, antibiofilm activity and modulation of virulence-associated processes. By comparison, *D. angolensis* represents a less extensively investigated species with preliminary antimicrobial evidence. Future studies integrating bioactivity-guided fractionation, metabolomic profiling and mechanistic microbiological approaches will be essential to establish compound–activity relationships and evaluate the translational potential of both species.

### 6.4. Cytotoxic and Antiproliferative Activity

Selected preparations of both *D. trifasciata* and *D. angolensis* have exhibited in vitro cytotoxic or antiproliferative activity against human cancer cell lines, although the findings are preparation- and model-dependent and negative results have also been reported. Currently available studies indicate that these effects include differential cytotoxicity toward malignant and non-malignant cells, induction of apoptosis and, in some models, modulation of cell-cycle progression.

The cytotoxic potential of *D. trifasciata* has been investigated in several human cancer cell lines, including colorectal, pancreatic, lung, oesophageal and rhabdomyosarcoma models. Some extracts exhibited greater cytotoxicity toward malignant cells than toward non-malignant cells, although selectivity was not consistently assessed across studies [11]. Caspase-3/7 activation was reported in selected cell models, supporting apoptosis-associated cell death, although the responsible extract constituents and upstream molecular targets were not established [77]. Phytochemical analyses further revealed differences in phenolic composition among plant organs and extract fractions; however, the contribution of individual phenolic constituents to differences in cytotoxic potency was not directly demonstrated [37].

Additional evidence for the antiproliferative activity of *D. trifasciata* was reported by De Guzman [60], who evaluated a semi-purified extract against HCT116 colorectal carcinoma cells. The extract showed only weak activity, with IC_50_ values > 100 µg/mL in all three trials, compared with approximately 0.80–0.89 µg/mL for doxorubicin. Although the study was limited to a single cancer cell line and did not investigate molecular mechanisms, it provides important evidence of limited antiproliferative potency in this experimental model.

Comparative studies further indicate that cytotoxic activity varies among *Dracaena* species. Although *D. trifasciata* was less active than *D. suffruticosa* in some experimental models, molecular docking analyses predicted favourable interactions between several *D. trifasciata* phenolic constituents and epidermal growth factor receptor (EGFR). These computational findings are hypothesis-generating and do not demonstrate EGFR-mediated cytotoxicity or establish that purification would enhance biological activity [35].

Mechanistic evidence for *D. angolensis* is currently limited but particularly informative. Rhizome extracts induced G2/M cell-cycle arrest in PC-3 prostate cancer cells and promoted apoptosis accompanied by upregulation of the pro-apoptotic genes *TP53* and *BAX* together with downregulation of the anti-apoptotic gene *BCL2*. These changes are consistent with involvement of apoptosis-related signalling, although they do not by themselves establish activation of the intrinsic mitochondrial apoptotic pathway [14].

Additional extract-level evidence for *D. angolensis* showed concentration-dependent cytotoxicity of the crude extract toward MCF-7, HeLa and SKOV3 cells, with substantially lower cytotoxicity toward non-malignant HUVEC cells [78].

Despite these findings, several limitations currently restrict interpretation of the available evidence. Most investigations have evaluated crude or partially purified extracts, making it difficult to identify the metabolites directly responsible for the observed cytotoxic effects. Moreover, substantial methodological differences in extraction procedures, plant organs and experimental models complicate direct comparison of reported IC_50_ values across studies. Notably, methanolic leaf and rhizome extracts of both *D. trifasciata* and *D. angolensis* showed no significant apoptosis-inducing activity in A549 cells within the concentration ranges tested [61]. Negative or weak findings have also been reported at the compound level: selected homoisoflavonoids from *D. angolensis* showed low or no cytotoxicity against HeLa cells (IC_50_ > 80 µM), while steroidal saponin pairs isolated from *D. trifasciata* showed no significant antiproliferative activity in the same cell line [12,16].

Overall, current evidence supports in vitro cytotoxic and antiproliferative activity of selected preparations from both *D. trifasciata* and *D. angolensis*, but does not establish anticancer efficacy. Apoptosis-associated changes, cell-cycle modulation and differential cytotoxicity between malignant and non-malignant cells have been observed in selected experimental models, whereas compound-specific mechanisms remain insufficiently characterized. Future studies should prioritize bioactivity-guided isolation of active metabolites, molecular target validation, normal-cell selectivity, pharmacokinetic characterization, and appropriate in vivo studies before therapeutic potential can be reliably assessed.

### 6.5. Metabolic and Enzyme-Inhibitory Activity

Current evidence suggests that *D. trifasciata* and, to a lesser extent, *D. angolensis* exert effects on glucose-related metabolic endpoints in experimental models, including inhibition of carbohydrate-digesting enzymes, modulation of hepatic glucose metabolism and protection of pancreatic β-cells. In addition, glucose-lowering effects have been reported for *D. trifasciata* in experimental diabetic animal models. Although the available evidence remains preclinical, the findings indicate that several distinct metabolic processes may be affected rather than a single molecular target.

One of the best-characterized in vitro effects is inhibition of α-glucosidase, a key enzyme responsible for intestinal carbohydrate digestion. Bioactivity-guided fractionation of *D. trifasciata* leaf extracts identified an active ethyl acetate subfraction with significant α-glucosidase inhibitory activity. Molecular docking further predicted favourable interactions of (2S)-3′,4′-methylenedioxy-5,7-dimethoxyflavone with the enzyme active site; however, the contribution of this compound to the experimentally observed inhibition was not directly established [48]. Earlier studies likewise demonstrated α-glucosidase inhibition in polar leaf extracts; however, the contribution of individual phenolic constituents to this activity was not established [29].

A distinct enzyme-inhibitory effect has been described for *D. angolensis*, whose methanolic leaf fraction inhibited glucose-6-phosphatase activity by 57.9% at 100 µM [36]. This finding demonstrates in vitro inhibition of an enzyme involved in hepatic glucose metabolism but does not by itself establish reduced hepatic glucose production in vivo.

The strongest mechanistic evidence currently available concerns the cytoprotective effects of *D. trifasciata* on pancreatic β-cells. Methanolic leaf extracts protected STZ-treated MIN6 cells, and pharmacological perturbation experiments supported the involvement of NF-κB-related signalling; proteasome inhibition abolished the protective response and affected regulation of the anti-apoptotic gene *cIAP2* [9]. Importantly, exposure to TNF-α eliminated the cytoprotective effect and shifted the cellular response towards cytotoxicity, indicating that the cytoprotective response of *D. trifasciata* is strongly influenced by the inflammatory microenvironment. These findings further suggest an important functional link between metabolic regulation and inflammatory signalling in this cellular model.

Overall, available evidence indicates that *D. trifasciata* and *D. angolensis* affect several glucose-related endpoints in preclinical models, including enzyme inhibition, pancreatic β-cell cytoprotection and, for *D. trifasciata*, glucose lowering in diabetic animals. However, these findings do not establish clinical antidiabetic efficacy, and further studies are required to identify the active metabolites, validate molecular mechanisms, characterize exposure and bioavailability, and determine whether the observed effects have therapeutic relevance in diabetes and metabolic disorders.

### 6.6. Other Biological Activities

Beyond the major pharmacological activities discussed above, *D. trifasciata* and *D. angolensis* have been investigated in several additional biological contexts, although the available evidence remains comparatively limited.

Preliminary studies suggest that *D. trifasciata* possesses analgesic and antipyretic activity. Ethanolic leaf extracts reduced nociceptive responses in several experimental pain models and attenuated yeast-induced pyrexia in mice, providing pharmacological support for analgesic and antipyretic effects in these experimental models [50]. However, these findings have not yet been extensively validated in independent experimental studies.

Enzyme-inhibitory activity beyond glucose-related targets has also been reported for *D. trifasciata*. Flavonoid-containing leaf fractions inhibited xanthine oxidase in vitro, with inhibition of 85.48% reported for an isolated fraction in one study and 60.27% for an extract obtained under optimized ultrasound-assisted enzymatic extraction conditions in another [79,80]. These findings demonstrate xanthine-oxidase inhibitory activity in vitro but do not establish antihyperuricemic or antigout efficacy in vivo.

Antimutagenic activity has also been reported for both species. In an Ames assay using sodium-azide-induced mutagenesis in *Salmonella typhimurium* TA100, rhizome extracts of *D. trifasciata* and *D. angolensis* significantly reduced the number of induced revertants at concentrations of ≥0.5 mg/plate, with the *D. angolensis* rhizome extract showing 76% antimutagenic activity at 1 mg/plate [81]. These findings provide in vitro antimutagenicity evidence but do not establish genoprotective efficacy in vivo.

Preliminary neuroprotective evidence has also been reported for *D. angolensis*. In a Wistar rat study evaluating hydro-alcoholic *S. cylindrica* leaf extract, treatment was associated with reduced escape latency in a behavioural memory task, while antioxidant and acetylcholinesterase-related endpoints were also investigated. Because several reported outcomes involved combinations with *Plumeria obtusa*, only effects separately attributable to *S. cylindrica* should be interpreted as species-specific evidence [82].

Potential nephroprotective activity has also been proposed for *D. trifasciata*. However, the available evidence is based primarily on secondary literature and in silico predictions rather than direct experimental studies of renal injury. Accordingly, nephroprotective activity and the proposed antioxidant, anti-inflammatory and cytoprotective mechanisms should currently be regarded as hypotheses requiring experimental validation [83].

Larvicidal activity against *Aedes aegypti* has also been evaluated for *D. trifasciata*. An ethanolic leaf extract produced only modest mortality of third-instar larvae, reaching 33.33% after 24 h and 40.00% after 48 h at the highest concentration tested [84]. These findings provide preliminary evidence of larvicidal activity, but the observed effect was limited under the experimental conditions used.

## 7. Molecular Mechanisms and Mechanistic Hypotheses

The experimental findings summarized in Section 6 have prompted investigation of the molecular mechanisms that may underlie selected biological effects of *D. trifasciata* and *D. angolensis*. Current mechanistic evidence varies substantially in strength, ranging from experimentally measured changes in molecular markers and pharmacological perturbation studies to computationally predicted ligand–target interactions. The following sections therefore distinguish experimentally supported molecular responses from mechanistic hypotheses that remain to be validated.

### 7.1. Nrf2/ARE-Associated Antioxidant Responses

Among the molecular mechanisms identified to date, Nrf2/ARE-associated responses represent one of the better-supported molecular observations related to the biological activity of *D. trifasciata*. In a thioacetamide-induced liver fibrosis model, Raslan et al. [20] demonstrated that root extracts significantly reduced oxidative liver injury, decreased serum ALT, AST and MDA levels, restored hepatic GSH content and increased the expression of *Nrf2*, *HO-1*, *NQO1* and *Keap1*. The root extract consistently produced stronger hepatoprotective effects than the corresponding leaf extract, and these molecular changes are consistent with involvement of Nrf2/ARE-associated antioxidant responses; however, direct pathway activation and its causal contribution to the hepatoprotective effect were not demonstrated.

Despite these encouraging findings, the phytochemicals directly responsible for the observed Nrf2/ARE-associated responses remain unidentified. Based on the current knowledge of plant polyphenols and electrophilic phytochemicals, modification of Keap1-Nrf2 interactions represents one possible general mechanism by which phytochemicals can influence Nrf2 signalling; however, this mechanism has not been directly investigated for extracts or isolated metabolites of *D. trifasciata* or *D. angolensis*. Whether steroidal saponins, flavonoids or phenolic acids represent the principal contributors to the observed Nrf2/ARE-associated responses in *D. trifasciata* has not yet been experimentally established. Consequently, identification of the active metabolites responsible for these molecular effects, together with direct validation of Nrf2/ARE pathway involvement, remains an important objective for future mechanistic studies.

### 7.2. NF-κB-Related Inflammatory and Cytoprotective Signalling

Current evidence identifies NF-κB signalling as one of the experimentally investigated signalling pathways potentially involved in the biological activity of *D. trifasciata*. Methanolic leaf extracts protected STZ-treated MIN6 pancreatic β-cells, and pharmacological perturbation experiments supported the involvement of NF-κB-related signalling, as inhibition of proteasome activity with MG132 abolished the cytoprotective response and prevented regulation of the anti-apoptotic gene *cIAP2* [9]. These findings support an association between NF-κB-related signalling and pancreatic β-cell protection, although direct pathway activation and causal involvement of specific extract constituents were not demonstrated.

Importantly, the biological effects of *D. trifasciata* appear to depend strongly on the inflammatory microenvironment. Co-treatment with TNF-α abolished the protective activity of the extract and shifted the cellular response toward cytotoxicity, supporting the interpretation that the observed cytoprotective response is highly context-dependent rather than uniformly anti-inflammatory or cytoprotective. This observation indicates that inflammatory conditions may substantially modify the cellular response to the extract; however, the relevance of this interaction to diabetes or chronic inflammatory disorders requires validation in appropriate in vivo models.

The involvement of MAPK signalling has been proposed based on the known biological activities of structurally related flavonoids and phenolic compounds. However, direct experimental evidence demonstrating modulation of ERK, JNK or p38 signalling by extracts or isolated metabolites of *D. trifasciata* or *D. angolensis* is currently lacking. Consequently, MAPK signalling should presently be regarded as a putative rather than experimentally validated mechanism requiring further investigation.

### 7.3. Apoptosis and Cell-Cycle Regulation

Apoptosis-associated responses represent one of the experimentally supported mechanisms underlying the in vitro cytotoxic effects of extracts from *D. trifasciata* and *D. angolensis*. Current evidence indicates that both *D. trifasciata* and *D. angolensis* can induce apoptosis-associated changes in selected cancer cell models, although the strongest mechanistic support is currently available for apoptosis and cell-cycle regulation in *D. angolensis*.

Rhizome extracts of *D. angolensis* induced G2/M cell-cycle arrest together with increased expression of the pro-apoptotic genes *TP53* and *BAX* and reduced expression of the anti-apoptotic gene *BCL2* in PC-3 prostate cancer cells [14]. This coordinated shift in apoptosis-related gene expression is consistent with involvement of the intrinsic mitochondrial apoptotic pathway. Nevertheless, definitive confirmation remains unavailable because key mitochondrial events, including cytochrome *c* release, mitochondrial membrane depolarization and caspase-9 activation, were not directly investigated.

Apoptosis induction has also been demonstrated for *D. trifasciata*. Leaf extracts activated executioner caspases-3/7 in rhabdomyosarcoma and oesophageal squamous carcinoma cells, providing evidence of apoptosis-associated cell death, although the upstream signalling events remain unresolved [77].

Evidence at the level of individual metabolites remains comparatively limited. Among the steroidal saponins isolated from *D. trifasciata*, the trifasciatoside K/L and M/N pairs showed no significant antiproliferative activity against HeLa cells [16]. These findings demonstrate that antiproliferative activity cannot be assumed from the presence of steroidal saponins alone and reinforce the need for compound-specific biological evaluation.

Despite increasing evidence for apoptosis-mediated cytotoxicity, the metabolites directly responsible for these effects have not yet been unequivocally identified through bioactivity-guided isolation. Establishing clear compound-target relationships therefore remains one of the principal challenges for future mechanistic studies of the cytotoxic and antiproliferative effects of *Dracaena*-derived preparations.

### 7.4. Quorum-Sensing and Virulence-Associated Mechanisms

In addition to direct bacterial growth inhibition, experimental evidence indicates that *D. trifasciata* preparations can affect virulence-associated processes in *Pseudomonas aeruginosa*. Bioactivity-guided studies demonstrated inhibition of biofilm formation by an active leaf fraction, with approximately 60% biofilm inhibition observed at the minimum inhibitory concentration (MIC, 4 mg/mL) [40]. Structural alterations of bacterial cells were also observed by scanning electron microscopy; however, these findings do not by themselves establish a specific molecular mechanism.

More direct mechanistic evidence was obtained from analysis of quorum-sensing-associated virulence responses. Treatment with an active *D. trifasciata* fraction reduced extracellular protease activity by 77.1% and decreased the relative expression of the *lasA*, *lasB*, *lasI*, *lasR*, *rhlI*, and *rhlR* genes to 0.81, 0.93, 0.76, 0.97, 0.90, and 0.55, respectively, compared with the untreated control [55]. These findings provide experimental evidence that the fraction affects quorum-sensing-associated virulence regulation, particularly the las and rhl systems. However, because the study evaluated a phytochemically complex fraction rather than isolated compounds, the metabolites responsible for these effects and their direct molecular targets remain unidentified.

Chemical profiling of antibacterially active fractions has identified several candidate constituents, including terpenoid- and phenolic-related metabolites. Nevertheless, detection of a compound in an active fraction does not establish that it is responsible for quorum-sensing or antibiofilm activity. Compound-specific validation, including testing of isolated metabolites against defined quorum-sensing targets and virulence endpoints, is therefore required to establish causal compound-mechanism relationships.

Overall, quorum-sensing and virulence-associated effects represent one of the more directly supported mechanistic observations for the antimicrobial activity of *D. trifasciata*, because changes in specific virulence-related gene expression and protease endpoints have been experimentally measured. However, the evidence remains limited to in vitro bacterial models, and comparable mechanistic studies have not yet been reported for *D. angolensis*.

### 7.5. Metabolic Targets and Enzyme Inhibition

Maintenance of glucose homeostasis requires coordinated regulation of intestinal carbohydrate digestion, hepatic glucose production, pancreatic β-cell survival and oxidative stress. Current evidence indicates that *D. trifasciata* and *D. angolensis* affect several glucose-related endpoints in experimental models, although the available evidence does not establish a unified multitarget antidiabetic mechanism.

One of the principal experimentally demonstrated enzyme-inhibitory effects for *D. trifasciata* is inhibition of α-glucosidase, an enzyme involved in intestinal carbohydrate digestion. Bioactivity-guided fractionation demonstrated α-glucosidase inhibition in polar leaf extracts [29], while molecular docking analyses further predicted interactions between selected flavonoid constituents and α-glucosidase, although definitive compound-level validation remains unavailable [29,48].

A complementary experimental observation involves cytoprotection of pancreatic β-cells. As discussed in Section 6.5, *D. trifasciata* extracts protected STZ-treated MIN6 cells and pharmacological perturbation experiments supported the involvement of NF-κB-related signalling, indicating that maintenance of β-cell survival may represent an additional component of glucose regulation [9].

Evidence from in vivo studies further supports a broader metabolic effect. In STZ-induced diabetic rats, *D. trifasciata* treatment reduced blood glucose and was accompanied by reductions in oxidative, apoptotic and vascular inflammatory markers [8], suggesting that glucose-lowering effects may occur alongside cytoprotective and anti-inflammatory responses, although causal relationships among these effects have not been established.

In contrast, *D. angolensis* showed glucose-6-phosphatase inhibition in a cell-free enzyme assay, with the methanolic fraction producing 57.9% inhibition at 100 µM [36]. Because this effect has not been validated as a mechanism of glucose lowering in vivo, it should currently be interpreted as enzyme-inhibitory evidence rather than as a demonstrated mechanism of hepatic glucose regulation.

Overall, the available evidence indicates effects on several glucose-related targets and endpoints, including α-glucosidase and glucose-6-phosphatase inhibition, pancreatic β-cell cytoprotection and glucose lowering in experimental diabetic animals. However, the relative contribution of each process to the observed metabolic effects remains unknown, and future studies should clarify whether these pathways are causally linked and whether the enzyme-inhibitory and cellular findings translate to physiologically relevant effects in vivo.

### 7.6. Computationally Predicted Mechanisms

Among the mechanisms proposed for *D. trifasciata*, anti-alopecia activity has attracted increasing attention owing to the potential interaction of its phytochemicals with androgen signalling pathways. Because androgenetic alopecia is driven primarily by dihydrotestosterone (DHT)-mediated activation of androgen receptors, modulation of androgen receptor signalling or inhibition of 5α-reductase represents a potential molecular strategy for investigation. Experimental evidence includes an in vivo rabbit hair-growth study in which a bioactive subfraction of *S. trifasciata* promoted hair growth and was associated with an anagen:telogen ratio of 78.26:21.79%, with hair-growth effects reported as comparable to 2% minoxidil under the conditions tested [85]. However, the molecular mechanism responsible for this effect was not established.

Mechanistic investigation of this potential activity has focused primarily on computational analyses [86,87], supported in some studies by chemical profiling of the investigated fractions [86]. LC-MS/MS analysis of *D. trifasciata* leaf fractions annotated several candidate metabolites, including methyl pyropheophorbide A, oliveramine, (2S)-3′,4′-methylenedioxy-5,7-dimethoxyflavane and methyl gallate [86]. Subsequent virtual screening, molecular docking, molecular dynamics and MM-PBSA analyses predicted favourable interactions of selected metabolites, including methyl pyropheophorbide A, with the androgen receptor [86]. These findings generate the hypothesis that androgen-receptor interactions may contribute to the potential trichological effects of *D. trifasciata*. Inhibition of 5α-reductase has also been proposed as a complementary mechanism capable of reducing DHT production; molecular docking and dynamics studies subsequently prioritized compounds including oliveramine, for which a docking score of −9.14 kcal/mol was reported [88]. However, 5α-reductase inhibition by these compounds has not been experimentally demonstrated.

Computational approaches have also been used to propose metabolic molecular targets for *D. trifasciata*. Docking and molecular-dynamics analyses predicted interactions of selected plant-derived compounds with hexokinase 2 (HK2) and glutaminase 1 (GLS1), with reported docking scores of approximately −8.19 and −8.99 kcal/mol, respectively [89]. These findings should likewise be regarded as target-prediction evidence rather than demonstration of enzyme inhibition or metabolic activity.

Although an in vivo hair-growth effect has been reported, the molecular mechanisms proposed for this activity remain computationally derived. No experimental studies have yet confirmed androgen-receptor binding, 5α-reductase inhibition by the computationally prioritized compounds, inhibition of DHT synthesis, or a causal relationship between these predicted molecular targets and the observed hair-growth response. Similarly, the predicted HK2 and GLS1 interactions require biochemical and cellular validation before they can be considered metabolically relevant targets.

The anti-alopecia potential of *D. trifasciata* is therefore supported by preliminary in vivo phenotypic evidence, whereas the proposed androgen-receptor- and 5α-reductase-related mechanisms remain computational hypotheses requiring experimental validation. Future studies should combine compound isolation with direct target-binding or enzyme-inhibition assays, cellular assays, follicle organ culture and well-controlled in vivo models to determine whether the predicted receptor interactions translate into measurable biological effects.

## 8. Safety and Toxicological Evidence

Safety data for *D. trifasciata* and *D. angolensis* remain substantially less developed than evidence concerning their biological activities. Available studies include single-dose acute investigations for both species and limited 28-day repeated-dose evidence for *D. trifasciata*; however, the evidence base remains small and heterogeneous with respect to extract composition, dose, sex, endpoints and study design. Evidence from longer-term subchronic or chronic studies, as well as genotoxicity, reproductive and developmental toxicity and herb-drug interaction studies, remains insufficient or unavailable.

For *D. trifasciata*, acute toxicity has been evaluated using several extract preparations. Ighodaro et al. [90] evaluated a leaf extract in Wistar rats and reported an intraperitoneal LD_50_ of 774.6 mg/kg, whereas oral administration at 18,000 mg/kg caused no mortality or reported adverse behavioural changes during the 24 h observation period, corresponding to an oral LD_50_ > 18,000 mg/kg. The marked difference between oral and intraperitoneal exposure emphasizes the importance of administration route when interpreting toxicity estimates.

Additional acute oral safety evidence was obtained for a chloroform leaf extract of *D. trifasciata* in female Wistar rats using an OECD 420-based protocol. A single oral dose of 2000 mg/kg followed by a 14-day observation period caused no mortality or detectable adverse effects in the measured clinical, behavioural, hematological, biochemical or relative-organ-weight endpoints [91]. These findings indicate acute tolerability of this specific preparation under the conditions tested but do not establish safety during prolonged exposure.

Earlier acute toxicity testing of ethanolic and aqueous leaf extracts of *D. trifasciata* in mice yielded LD_50_ values of 1513.5 ± 21.5 mg/kg and 1426 ± 43.6 mg/kg, respectively [50]. Differences in extract composition, experimental design and administration procedures across these studies limit direct comparison of the reported toxicity estimates.

Repeated-dose evidence for *D. trifasciata* is available but remains limited. Fitria et al. [92] administered a chloroform leaf extract orally to Wistar rats at 1000 mg/kg/day for 28 consecutive days using an OECD 407-based design. No mortality or significant differences from vehicle or placebo controls were reported for the monitored clinical and physiological endpoints, including behaviour, body weight, body temperature, food and water intake, fecal condition and fasting blood glucose. However, the relatively restricted endpoint panel limits conclusions regarding systemic repeated-dose toxicity.

Safety information for *D. angolensis* is similarly limited. In an acute oral toxicity study, a hydro-alcoholic preparation administered to female Wistar rats at 2000 mg/kg caused no mortality or major clinical toxicity during the 14-day observation period [93]. These findings provide acute limit-dose tolerability evidence for the tested preparation rather than evidence of general safety of *D. angolensis*.

Importantly, the available literature remains insufficient to characterize longer-term subchronic or chronic toxicity, genotoxicity, reproductive or developmental toxicity, toxicokinetics, or clinically relevant herb–drug interactions for either *D. trifasciata* or *D. angolensis*. The existing studies also employ different preparations and dose regimens, preventing establishment of generalized safe exposure levels or direct extrapolation to human therapeutic use.

Overall, the available toxicological evidence indicates relatively limited acute toxicity for several orally administered preparations and provides preliminary 28-day repeated-dose data for *D. trifasciata*. However, the small number and heterogeneity of studies, together with the absence of comprehensive longer-term and specialized toxicological assessment, preclude establishment of a complete safety profile. Future studies should prioritize standardized repeated-dose, subchronic and chronic toxicity testing of chemically characterized preparations, together with genotoxicity, reproductive and developmental toxicity, toxicokinetic evaluation, target-organ assessment and investigation of potential herb-drug interactions.

## 9. Emerging Non-Pharmacological Applications

The following applications fall outside the core pharmacological evidence synthesis and are therefore discussed separately as emerging non-pharmacological uses of *D. trifasciata* and *D. angolensis*.

### 9.1. Green Nanotechnology and Nanoparticle Synthesis

*D. trifasciata* and *D. angolensis* have recently attracted attention as biological resources for green synthesis of functional nanoparticles. Extracts from these species have been used as reducing and stabilizing agents in plant-mediated nanoparticle synthesis [94,95]. Plant-mediated approaches have been applied to silver nanoparticles, selenium nanoparticles, ZnO nanoparticles and Ag-doped ZnO nanocomposites [94,95,96]. Although most studies have focused primarily on nanoparticle synthesis and physicochemical characterization, only a few have evaluated their biological properties.

Among these, biosynthesized silver nanoparticles prepared from *D. trifasciata* leaf extracts exhibited radical-scavenging activity in a chemical antioxidant assay, whereas no significant anti-inflammatory activity was observed in a protein denaturation assay [97]. Because the activity of the corresponding parent extract was not evaluated under directly comparable conditions, these findings do not establish whether nanoparticle formation preserved or modified the biological properties of the plant preparation. Consequently, the biomedical relevance of *Dracaena*-derived nanoparticles remains uncertain.

At present, the principal limitation of this field is the scarcity of standardized biological investigations. Nanoparticle activity is strongly influenced by particle size, morphology, surface chemistry, stability and the composition of the phytochemical corona, making direct comparison between studies difficult. Future research should therefore integrate comprehensive physicochemical characterization with standardized in vitro and in vivo biological evaluation, toxicological assessment and cellular uptake analyses before any biomedical advantages over the corresponding plant extracts can be established.

### 9.2. Cellulose Fibres and Biomaterial Applications

Another emerging application of *Dracaena* species involves the use of cellulose-rich fibres as sustainable biomaterials. This field is particularly relevant to *D. angolensis*, whose leaves and floral stalks provide fibres with favourable structural and mechanical properties. Although current research has been driven primarily by materials science, these fibres have also been investigated as candidates for selected biomedical applications, including surgical suture materials.

Several studies have demonstrated that *D. angolensis* fibres possess high cellulose content, crystallinity and favourable thermal stability, providing a physicochemical basis for further biomaterial investigation [98]. Chemical modification, particularly alkali treatment, has been reported to improve fibre purity and selected physicochemical and mechanical properties through removal of non-cellulosic components [99,100]. Similar cellulose enrichment following alkali treatment has also been reported for *D. trifasciata* [101].

Among the studies with direct biomedical relevance, Kampeerapappun et al. [102] evaluated purified cellulose fibres derived from *D. angolensis* as candidate non-absorbable surgical suture materials. Fibre purification increased cellulose content and improved selected physicochemical properties relevant to suture development. Importantly, the study also included an in vivo biocompatibility assessment, in which host-tissue reactions did not differ significantly from those observed with a commercial braided silk suture. These findings provide preliminary biological, in addition to physicochemical, support for this specific application.

Nevertheless, the biomedical evidence remains limited to a single preclinical investigation, and the available literature on *Dracaena*-derived fibres is otherwise dominated by physicochemical and materials-science characterization. Broader evaluation of cytocompatibility, sterilization stability, longer-term tissue responses, infection risk, biodegradation and performance in wound-healing models is therefore required before clinical suitability can be established. Consequently, *Dracaena*-derived fibres should currently be regarded as candidate biomaterials rather than validated biomedical materials.

Future research should integrate standardized biomaterial characterization with comprehensive biological evaluation, including cytotoxicity testing, biodegradation, tissue integration and in vivo wound-healing studies, to determine whether the favourable physicochemical properties of *Dracaena* fibres can be translated into biologically compatible and functionally suitable biomaterials.

### 9.3. Indoor-Air Phytoremediation

*D. trifasciata* has also been investigated in the context of indoor-air pollutant removal. A recent literature review summarized experimental reports concerning carbon monoxide, formaldehyde and other indoor pollutants but emphasized substantial heterogeneity in plant preparations, experimental systems and testing conditions [103]. Accordingly, this environmental application remains outside the core pharmacological evidence synthesis and requires standardized validation under realistic indoor conditions before practical effectiveness can be established.

## 10. Research Gaps and Future Priorities

Despite considerable progress in the phytochemical and pharmacological characterization of *D. trifasciata* and *D. angolensis*, several key knowledge gaps continue to limit their translational development.

The most important limitation concerns the incomplete identification of bioactive metabolites responsible for the observed biological effects. Although steroidal saponins, homoisoflavonoids, flavonoids and phenolic acids have been identified in preparations showing biological activity, only a small number of compounds have been investigated individually. Consequently, bioactivity-guided fractionation and compound-level validation should become a priority for future research. The widespread reliance on crude or partially purified extracts currently limits the establishment of robust compound–activity and structure–activity relationships.

A second major challenge is the limited mechanistic validation of the proposed molecular targets. Among the signalling pathways discussed in the present review, Nrf2/ARE-associated responses are supported by in vivo changes in oxidative-stress and gene-expression markers, although direct pathway activation and causal involvement have not been demonstrated. NF-κB involvement is supported primarily by pharmacological perturbation experiments in cellular models, while involvement of MAPK signalling remains hypothetical. Similarly, apoptosis-related mechanisms have been associated with gene-expression changes and caspase activation, but direct confirmation of mitochondrial signalling events remains unavailable. Quorum-sensing-associated effects are supported by changes in *las*/*rhl* gene expression and virulence-related endpoints, although the responsible metabolites and direct molecular targets remain unidentified. Computational predictions involving androgen receptor, 5α-reductase, HK2 and GLS1 remain hypothesis-generating and require experimental validation. Future studies should therefore combine molecular, cellular and animal models to establish causal relationships between individual phytochemicals and specific signalling pathways.

Translation of these findings into therapeutic applications is further limited by the scarcity of pharmacokinetic and toxicological studies. Information regarding bioavailability, metabolism, tissue distribution and long-term safety of *Dracaena*-derived metabolites is almost completely lacking. Available toxicological evidence includes acute and limited 28-day repeated-dose studies; however, longer-term subchronic and chronic toxicity, genotoxicity, reproductive and developmental toxicity, toxicokinetic data, and clinically relevant herb-drug interaction studies remain insufficient or unavailable. Such data will be essential before these compounds can be considered as potential drug candidates.

Another important limitation is the lack of methodological standardization. Considerable variability in plant material, cultivar, extraction procedures and experimental protocols currently hampers comparison among studies. Future investigations should integrate standardized phytochemical profiling with harmonized biological assays and transparent reporting of experimental conditions. Particular attention should be given to botanical authentication, plant origin and voucher documentation, extract standardization, tested dose or concentration, appropriate comparators, and quantitative reporting of biological endpoints.

Several emerging applications, including green nanotechnology and biomaterials, remain at an early stage of development. Green-nanotechnology studies are dominated by nanoparticle synthesis and physicochemical characterization, with comparatively limited biological evaluation, whereas biomaterial research includes preliminary in vivo biocompatibility evidence for *D. angolensis*-derived cellulose fibres as candidate surgical sutures. Nevertheless, broader biological and preclinical validation remains necessary before the practical or biomedical relevance of these applications can be established. Indoor-air phytoremediation likewise requires validation under environmentally relevant real-world conditions. Preliminary in vivo anti-alopecia evidence requires independent replication and mechanistic clarification, whereas the proposed androgen-receptor and 5α-reductase interactions remain supported by computational analyses. Progress in these areas will require systematic evaluation using application-appropriate experimental models.

Finally, greater integration of ethnopharmacology with modern phytochemistry and molecular pharmacology may provide a more efficient strategy for identifying experimentally relevant bioactive compounds. Comparative investigation of traditional preparations and laboratory-derived extracts may help bridge the current gap between empirical medicinal use and experimental pharmacological evidence. In particular, direct comparison of traditional aqueous preparations with the ethanolic or methanolic extracts commonly used in experimental studies would help determine whether existing pharmacological findings are relevant to documented ethnomedicinal practices.

Overall, future research should move beyond descriptive phytochemical characterization toward mechanism-driven, compound-oriented and translational studies. Such an approach will be essential to determine which of the currently reported biological activities are reproducible, compound-specific, mechanistically validated and sufficiently supported by pharmacokinetic and safety evidence to justify further translational development.

## 11. Conclusions

This review integrates current knowledge on the phytochemistry, ethnopharmacology, pharmacological activities, safety, and molecular mechanisms of *Dracaena trifasciata* and *D. angolensis*, highlighting their structural diversity as sources of bioactive plant-derived metabolites. Both species contain structurally diverse phytochemicals-including steroidal saponins, homoisoflavonoids, flavonoids and phenolic acids- and their extracts, fractions, or selected isolated compounds have shown chemical antioxidant capacity, anti-inflammatory and antimicrobial effects, enzyme-inhibitory activity, metabolic effects in animal models, and in vitro cytotoxic or antiproliferative activity. However, the contribution of individual metabolites to many of these effects remains unresolved.

Among the molecular mechanisms identified to date, Nrf2/ARE-associated responses are supported by changes in oxidative-stress and gene-expression markers in an in vivo model, whereas evidence for NF-κB modulation is based primarily on pharmacological perturbation experiments in cellular models. Apoptosis- and cell-cycle-related changes and quorum-sensing-associated virulence regulation have also been experimentally observed in selected models. Other proposed mechanisms, including MAPK signalling and computationally predicted molecular targets, require substantially stronger experimental validation before firm conclusions can be drawn.

The available evidence also demonstrates a clear imbalance in current research. *D. trifasciata* is considerably better characterized, with multiple studies describing its phytochemical composition and biological activities across chemical, enzymatic, cellular and animal models. In contrast, *D. angolensis* remains comparatively underexplored despite its distinctive phytochemical profile and available preliminary biological and pharmacological evidence. Expanding phytochemical characterization and mechanistic investigation of this species therefore represents an important research priority.

A recurring limitation throughout the literature is the predominant use of crude or partially characterized plant extracts, which hinders identification of the metabolites responsible for the observed biological effects and limits the establishment of structure–activity relationships. Future studies should therefore prioritize bioactivity-guided isolation of active constituents, standardized phytochemical characterization, comprehensive pharmacokinetic and toxicological evaluation, and validation of molecular mechanisms in physiologically relevant experimental models. In particular, current safety evidence is limited to a small number of acute and 28-day repeated-dose studies and remains insufficient to establish long-term safety or safe human exposure levels.

Overall, the available evidence supports *D. trifasciata* and *D. angolensis* as sources of structurally diverse bioactive metabolites, with experimentally documented chemical antioxidant capacity, antimicrobial and anti-inflammatory effects, enzyme-inhibitory and metabolic activities, and in vitro cytotoxic or antiproliferative effects, depending on the preparation and experimental model. Although the current evidence is strongest for *D. trifasciata*, expanding mechanistic and phytochemical investigation of *D. angolensis* represents an important opportunity for future research. Continued integration of phytochemistry, molecular pharmacology, pharmacokinetics, and safety assessment will be essential to determine whether the observed preclinical activities can support further translational development.

## Figures and Tables

**Table 2:** Structural classes of steroidal glycosides identified in *Dracaena trifasciata* and their principal structural and experimentally evaluated biological characteristics.

Structural Class	Representative Compounds	Steroidal Skeleton	Characteristic Structural Features	Experimentally Reported Biological Activity	Reference
Diosgenin-type steroidal saponins	Trifasciatosides A–J	Spirostanol and furostanol	Glycosylated diosgenin derivatives differing in steroidal skeleton and glycosylation pattern	Compounds were isolated and structurally characterized; compound-specific biological activity was not evaluated in this study	[15]
Monoacetylated steroidal saponins	Trifasciatosides K/L	Spirostanol	Regioisomeric monoacetylated steroidal glycosides	No significant antiproliferative activity against HeLa cells for the tested pair	[16]
Hydroxylated monoacetylated steroidal saponins	Trifasciatosides M/N	Spirostanol	Additional hydroxylation compared with K/L; isolated as inseparable regioisomeric pairs	No significant antiproliferative activity against HeLa cells for the tested pair	[16]
Related phenolic metabolite	Trifasciatine C	Dihydrochalcone derivative	Dihydrochalcone derivative co-isolated with steroidal saponins	Biological activity not evaluated	[16]

**Table 3:** Comparative pharmacological and biological evidence for *Dracaena trifasciata* and *Dracaena angolensis*.

**Species**	**Plant Material/Preparation**	Experimental Model	Dose/Concentration	Comparator/Control	Main Quantitative Outcome	Evidence Level	Mechanism Status	Reference
**Metabolic and enzyme-inhibitory evidence**								
*D. trifasciata*	Leaf decoction	Alloxan-induced diabetic Wistar rats; pancreatic histology	100, 150, 200 mg/kg bw for 30 d	Glibenclamide 5 mg/kg; diabetic control	All three doses reduced blood glucose and increased pancreatic β-cell insulin-granule density relative to diabetic controls	ANIMAL	Not directly investigated	[47]
*D. trifasciata*	Plant extract	STZ-induced diabetic Wistar rats	50 and 100 mg/kg for 15 d	Glibenclamide 0.5 mg/kg; diabetic control	Day-15 fasting glucose: 80.22 ± 4.9 mg/dL at 100 mg/kg vs. 289.49 ± 9.8 mg/dL in STZ controls; reductions in TBARS, apoptosis, VEGF, ICAM-1, IL-6 and MCP-1 were also reported	ANIMAL	Associated with measured oxidative, inflammatory and apoptotic markers; causal pathway not established	[8]
*D. trifasciata*	Methanolic leaf extract	STZ-injured MIN6 pancreatic β-cells; RAW cells	5–15 mg/mL	STZ; TNF-α 100 ng/mL; MG132 ≥ 0.2 µM	Extract reduced STZ-associated toxicity; TNF-α and MG132 abolished the protective effect	CELL	NF-κB involvement indirectly supported by pharmacological perturbation; direct pathway activation not demonstrated	[9]
*D. trifasciata*	Ethyl-acetate fraction and subfractions	α-Glucosidase inhibition assay	100–500 µg/mL	Acarbose	IC_50_: fraction 170.82 µg/mL; subfraction C 183.47 µg/mL; subfraction E 74.89 µg/mL; acarbose 0.29 µg/mL	ENZ	Enzyme inhibition experimentally demonstrated; ligand–target interactions computationally predicted	[48]
*D. angolensis*	Ethanolic extract and solvent fractions	Rat-liver glucose-6-phosphatase assay	100 µM	Sodium orthovanadate 100 µM	Inhibition: ethanol extract 51.3%; methanol fraction 57.9%; sodium orthovanadate 53.4%	ENZ	Enzyme inhibition demonstrated; no in vivo glucose-lowering mechanism established	[36]
**Lipid metabolism and hepatoprotective evidence**								
*D. trifasciata*	Methanolic leaf extract	Diet-induced obese female Wistar rats; *n* = 6/group	100 or 200 mg/kg bw/day for 4 weeks	Obese untreated control	At 100 mg/kg: glucose −44.12%, TG −45.66%, FFA −53.36%; at 200 mg/kg: −45.55%, −48.79% and −63.82%, respectively	ANIMAL	Metabolic effects demonstrated in vivo; specific molecular mechanism not established	[49]
*D. trifasciata*	Ethanolic leaf extract	HFD-induced hyperlipidemia in Wistar rats; *n* = 6/group	200 and 400 mg/kg	Atorvastatin 10 mg/kg; HFD control	At 400 mg/kg: TC 125.62 ± 5.48 vs. 200.00 ± 3.41 mg/dL; TG 185.15 ± 4.93 vs. 320.57 ± 5.73 mg/dL; LDL-C 54.24 ± 2.17 vs. 117.91 ± 3.57 mg/dL	ANIMAL	Antihyperlipidemic effect demonstrated in an HFD model; molecular target not established	[10]
*D. trifasciata*	Leaf and root extracts	Thioacetamide-induced liver fibrosis in rats	100 and 200 mg/kg/day	TAA injury control	ALT, AST and MDA decreased; GSH and hepatic Nrf2, HO-1, NQO1 and Keap1 mRNA increased; root extract was more active	ANIMAL	Associated with Nrf2/ARE-related gene-expression changes; direct Nrf2 activation was not demonstrated	[20]
**Analgesic, antipyretic, anti-inflammatory and related evidence**								
*D. trifasciata*	Ethanolic and aqueous leaf extracts	Acetic-acid writhing, tail-immersion, formalin and yeast-induced pyrexia models	100 and 200 mg/kg	Model-specific reference drugs	Dose-related analgesic responses were observed; ethanolic extract at 200 mg/kg significantly reduced yeast-induced pyrexia	ANIMAL	Not directly investigated	[50]
*D. trifasciata*	Methanolic extract	Acetic-acid writhing, tail-immersion and formalin tests; *n* = 6/group	400 mg/kg	Diclofenac 10 mg/kg in writhing assay	Writhing inhibition: 54.6% vs. 82.2% with diclofenac; effects in tail-immersion and formalin tests were weak/non-significant	ANIMAL	COX/prostaglandin involvement proposed by authors but not experimentally measured	[51]
*D. trifasciata*	Ethanolic leaf extract	Acetic-acid writhing in mice; *n* = 5/group	250 and 500 mg/kg	Diclofenac 25 mg/kg	Writhing inhibition 33.63% and 44.98%, respectively, vs. 52.84% with diclofenac	ANIMAL	Not investigated	[52]
*D. trifasciata*	Ethanolic leaf extract	Experimental allergy/anaphylaxis models	100 and 200 mg/kg	Model-specific controls	Reduced eosinophilia/leukocytosis, mast-cell degranulation and cutaneous anaphylactic responses	ANIMAL	Mast-cell stabilization/mediator modulation indirectly supported; intracellular mechanism not established	[53]
*D. angolensis*	Hydro-alcoholic leaf extract	Membrane-stabilization and protein-denaturation assays	Concentration series	Reference anti-inflammatory agents	Dose-dependent cell-free anti-inflammatory activity was reported	CELL-FREE	No molecular anti-inflammatory mechanism demonstrated	[54]
**Antimicrobial, antibiofilm and quorum-sensing evidence**								
*D. trifasciata*	Ethanolic and petroleum-ether leaf extracts	Six bacterial species; agar diffusion and MIC	0.156–20 mg/mL	Tetracycline 0.05 mg/mL	MICs ranged from 0.156 to 5 mg/mL; *K. pneumoniae* and *B. subtilis* were among the more susceptible organisms	CELL	Antibacterial activity demonstrated in vitro; mechanism not investigated	[11]
*D. trifasciata*	Active leaf fraction	*P. aeruginosa* biofilm assay	MIC 4 mg/mL	—	Approximately 60% biofilm inhibition at the MIC	CELL	Biofilm-associated effect demonstrated; molecular target not established	[40]
*D. trifasciata*	Active leaf fraction	*P. aeruginosa* protease and quorum-sensing assays	—	—	Protease activity inhibited by 77.1%; relative expression of *lasA*, *lasB*, *lasI*, *lasR*, *rhlI* and *rhlR* decreased to 0.81, 0.93, 0.76, 0.97, 0.90 and 0.55, respectively	CELL	Experimentally associated with quorum-sensing/virulence regulation	[55]
*D. trifasciata*	Bioassay-guided leaf fraction/subfraction	*S. aureus*	—	Chloramphenicol	Active subfraction: 18.33 ± 0.58 mm inhibition zone vs. 24.67 ± 0.58 mm with chloramphenicol	CELL	Bacterial structural alterations observed; compound-specific causality not established	[56]
*D. trifasciata*	Methanol and hexane leaf extracts	*S. aureus* ATCC 6538 and *E. coli* ATCC 8739	200 µL/well; extract concentration NR	Positive control, identity NR	Methanol: 21 mm (*S. aureus*) and 16 mm (*E. coli*); hexane: 11 mm and no activity, respectively; positive control: 32 and 29 mm	CELL	Antibacterial screening only	[57]
*D. trifasciata*	95% ethanolic extract of aerial non-flowering parts	*S. aureus*, *B. subtilis*, *E. coli*, *S. typhimurium*, *A. flavus and C. albicans;* disc diffusion and MIC assays	Disc: 20 µL extract; MIC: 1–50 µL/mL	Gentamicin 10 µg; ketoconazole 5 µg; DMSO control	Inhibition zones: *S. aureus* 15.43 mm, *E. coli* 16.0 mm, *A. flavus* 26.93 mm and *C. albicans* 22.50 mm; *B. subtilis* and *S. typhimurium* were resistant; MIC: *A. flavus* 4.88 µg/mL and *C. albicans* 19.53 µg/mL	CELL	Antibacterial and antifungal activity demonstrated in vitro; molecular mechanism not investigated	[45]
*D. trifasciata*	Sequentially sonicated chloroform and ethanolic leaf extracts	*S. aureus* ATCC 25923; Kirby–Bauer, MIC and MBC assays	25 µL/disc; MIC/MBC concentration series 31.25–1000 mg/mL	Antibiotic positive control; sterile-water/medium controls	Inhibition-zone area: 73.3 ± 2.8 mm^2^ (chloroform) and 110 ± 10 mm^2^ (ethanol); MIC/MBC: 500/1000 mg/mL (chloroform) and 31.25/62.50 mg/mL (ethanol)	CELL	Antibacterial activity demonstrated in vitro; ethanolic extract more active, but compound-specific mechanism not established	[58]
*D. angolensis*	Ethanolic leaf extract	*S. aureus*, *E. coli* and *P. aeruginosa*	—	—	*S. aureus* inhibition zones ranged from 16.4 ± 0.95 to 22.5 ± 1.24 mm depending on strain; lower activity was observed against *P. aeruginosa* and *E. coli*	CELL	Not investigated	[19,59]
**Cytotoxic and antiproliferative evidence**								
*D. trifasciata*	Ethanolic leaf extract	HCT116 colorectal cancer and primary colon epithelial cells	7.8–1000 µg/mL; 24–72 h	5-FU 10 µg/mL	HCT116 IC_50_ 10.0–14.07 µg/mL; normal PCE IC_50_ 92.9–216.9 µg/mL	CELL	Selective in vitro cytotoxicity demonstrated; molecular anticancer mechanism not established	[11]
*D. trifasciata*	Semi-purified extract	HCT116 cells	—	Doxorubicin	Extract IC_50_ > 100 µg/mL in all trials vs. approximately 0.80–0.89 µg/mL for doxorubicin	CELL—negative/weak	No mechanism demonstrated	[60]
*D. trifasciata*	Root and leaf fractions	MDA-MB-231 cells	—	—	IC_50_ 89.83 µg/mL for roots and 77.6 µg/mL for leaves	CELL	In vitro cytotoxicity only	[37]
*D. angolensis*	Methanolic leaf/rhizome extracts	MCF-7, PC-3 and Caco-2 cells	Concentration-dependent	Assay-specific controls	Rhizome extract was more active; in PC-3 cells total apoptosis reached 19.1%; *TP53* and *BAX* increased 3.33- and 4.52-fold, while *BCL2* decreased to 0.313-fold	CELL	Associated with G2/M arrest and apoptosis-related *P53/BAX/BCL2* changes	[14]
*D. angolensis*	Isolated rhizome homoisoflavanones	HeLa cells	—	—	Selected compounds showed IC_50_ > 80 µM or no significant cytotoxicity	CELL—negative	No cytotoxic mechanism supported	[12]
*D. trifasciata*	Isolated trifasciatines/steroidal saponins	HeLa cells	—	5-FU	No significant antiproliferative effect for tested compounds/pairs	CELL—negative	Not applicable	[16,27]
*D. trifasciata* and *D. angolensis*	80% methanolic leaf and rhizome extracts	A549 human lung adenocarcinoma cells	Cytotoxicity: 10–100 µg/mL, 2 and 4 h; apoptosis: 50–300 µg/mL, 24 h	Camptothecin 20 µM (apoptosis assay)	Leaf and rhizome extracts of both species showed no significant acute cytotoxicity at 10–100 µg/mL and no significant increase in apoptotic cells at 50–300 µg/mL	CELL—negative	No apoptosis-inducing activity demonstrated in A549 cells under the tested conditions	[61]

Abbreviations: 5-FU, 5-fluorouracil; ALT, alanine aminotransferase; ANIMAL, in vivo animal evidence; AST, aspartate aminotransferase; BAX, BCL2-associated X protein; BCL2, B-cell lymphoma 2; bw, body weight; CELL, cellular or microbiological in vitro evidence; CELL-FREE, non-cellular biological assay; ENZ, cell-free enzyme-inhibition evidence; FFA, free fatty acids; GSH, glutathione; HFD, high-fat diet; HO-1, heme oxygenase-1; ICAM-1, intercellular adhesion molecule 1; LDL-C, low-density lipoprotein cholesterol; MCP-1, monocyte chemoattractant protein-1; MDA, malondialdehyde; MG132, proteasome inhibitor; MIC, minimum inhibitory concentration; NQO1, NAD(P)H quinone oxidoreductase 1; NR, not reported; PCE, primary colon epithelial cells; STZ, streptozotocin; TAA, thioacetamide; TBARS, thiobarbituric acid reactive substances; TC, total cholesterol; TG, triglycerides; TNF-α, tumour necrosis factor alpha; VEGF, vascular endothelial growth factor. Note: Cytotoxicity in cancer cell lines was classified as in vitro cytotoxic/antiproliferative activity rather than anticancer efficacy. Enzyme inhibition was not considered equivalent to antidiabetic efficacy. Mechanistic interpretations were classified according to the level of direct experimental support.

## Data Availability

Data generated during the literature review, including the study-level inventory of included primary studies and the complete database-specific search strategies, are provided in the Supplementary Materials (Tables S1 and S2). Additional information on the literature identification and study-selection process is provided in Section 1 and Figure 1.

## Supplementary Materials

The following supporting information can be downloaded at: https://www.mdpi.com/article/10.3390/molecules31193520/s1. Table S1: Study-level inventory of primary studies included in the evidence synthesis; Table S2: Complete database-specific literature search strategies.
